# Speed up Multi‐Scale Force‐Field Parameter Optimization by Substituting Molecular Dynamics Calculations with a Machine Learning Surrogate Model

**DOI:** 10.1002/cphc.202500353

**Published:** 2025-09-05

**Authors:** Robin Strickstrock, Alexander Hagg, Dirk Reith, Karl N. Kirschner

**Affiliations:** ^1^ Department of Engineering and Communication (DEC) Institute of Technology, Resource and Energy‐efficient Engineering (TREE) Bonn‐Rhein‐Sieg University of Applied Sciences 53757 Sankt Augustin Germany; ^2^ Fraunhofer Institute for Algorithms and Scientific Computing (SCAI) Schloss Birlinghoven 53757 Sankt Augustin Germany; ^3^ Department of Computer Science, Institute of Technology, Resource and Energy‐efficient Engineering (TREE) Bonn‐Rhein‐Sieg University of Applied Sciences 53757 Sankt Augustin Germany

**Keywords:** force‐field optimization, gradient‐based optimization, Lennard–Jones parameters, machine learning, neural networks

## Abstract

Molecular modeling plays a vital role in many scientific fields, ranging from material science to drug design. To predict and investigate the properties of those systems, a suitable force field (FF) is required. Improving the accuracy or expanding the applicability of the FFs is an ongoing process, referred to as force‐field parameter (FFParam) optimization. In recent years, data‐driven machine learning (ML) algorithms have become increasingly relevant in computational sciences and elevated the capability of many molecular modeling methods. Herein, time‐consuming molecular dynamic simulations, used during a multiscale FFParam optimization, are substituted by a ML surrogate model to speed‐up the optimization process. Subject to this multiscale optimization are the Lennard–Jones parameters for carbon and hydrogen that are used to reproduce the target properties: *n*‐octane's relative conformational energies and its bulk‐phase density. By substituting the most time‐consuming element of this optimization, the required time is reduced by a factor of ≈20, while retaining FFs with similar quality. Furthermore, the workflow used to obtain the surrogate model (i.e., training data acquisition, data preparation, model selection, and training) for such substitution is presented.

## Graphical abstract

Substituting molecular dynamics simulations by a neural network surrogate model to speed up multiscale force‐field parameter optimization and increase knowledge about the optimization objective.

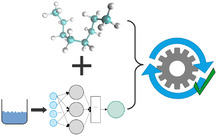

## 1 Introduction

Molecular modeling plays an essential role in many areas of biology and chemistry, for instance, material science and drug design.^[^ ^1^, ^2^ ^]^ Due to the atomistic level of detail and high temporal resolution, molecular mechanic (MM) and molecular dynamic (MD) methods provide insight into a wide range of microscopic phenomena. However, they are a simplification of reality–with the parameterized force fields (FFs) forming the foundation of these modeling methods, and whose generated data strongly depends on the used force‐field parameters (FFParams). While there are well‐known FF for biopolymers, in other systems (e.g., materials) those parameters are often determined on a case‐by‐case basis. Thus, there is a demand for improving FF accuracies and their application range. FF optimization often is a time‐consuming task that is prone to error and requires expert knowledge, with several assisting tools developed to aid the process. To name a few, there are the Open Force Field Initiative (OpenFF) evaluator^[^ ^3^ ^]^ developed by the OpenFF group;^[^ ^4^, ^5^, ^6^, ^7^ ^]^ LigParGen^[^ ^8^ ^]^ for OPLS‐AA;^[^ ^9^ ^]^ Paramfit,^[^ ^10^ ^]^ Parmscan,^[^ ^11^ ^]^ and Antechamber^[^ ^12^ ^]^ for AmberFF^[^ ^13^, ^14^ ^]^ (see the summary by He et al.^[^ ^15^ ^]^ for more information).

To bridge the gap between single molecular and ensemble properties, a modular, multiscale FFParam optimization toolkit called Force FieLd Optimization Workflow (FFLOW)^[^ ^16^, ^17^ ^]^ is under development, which can be seen as a successor of previous work in this area.^[^ ^18^, ^19^, ^20^ ^]^ In that work, it was shown that it is possible to simultaneously optimize FFParams toward target data originating from different property domains. This results in a set of optimized FFParams that are capable of reproducing the relative conformational energies (RCE) from MM minimizations and bulk‐phase density from MD simulations, using *n*‐octane as an example molecule. However, this iterative optimization workflow involved the evaluation of the targeted properties during each cycle, which resulted in time‐consuming optimization runs. Here, we aim to reduce the “real‐time to solution” required for optimization by substituting costly MD simulations with a machine learning (ML) surrogate model and establish a surrogate model‐assisted optimization (SMAOpt). For this purpose, we use the results of a previous optimization of *n−*octane's Lennard–Jones parameters (LJParams)^[^ ^17^ ^]^ as a reference and aim for comparable results regarding the accuracy of the optimization objective's reproduction (i.e., the bulk‐phase density and RCE). Furthermore, we present the workflow necessary to obtain the surrogate model for this substitution. A systematic comparison is performed on the different sampling strategies for constructing the training data, and the performance of various ML models (i.e., linear, polynomial, random forest, Gaussian process, and neural network regression).

There already exist several promising approaches for combining ML and FF optimization. For example, Fallani et al. created an inverse design to map certain properties to molecular structures;^[^ ^21^ ^]^ Wolinska et al. combined quality diversity (QD) algorithms and neural networks (NNs) to illuminate the property space in crystal structures;^[^ ^22^ ^]^ Deringer et al., Gokcan et al., and Anstine et al. used NNs to replace quantum mechanics (QM) calculations for molecular potential energies;^[^ ^23^, ^24^, ^25^ ^]^ Wang et al. used graph neural networks (GNNs) to obtain valence and nonbonded parameters for MM FFs;^[^ ^26^ ^]^ and Xie et al. used GNNs to predict electrostatic interactions in an ML/MM approach replacing a QM/MM workflow that is orders of magnitudes more time‐consuming.^[^ ^27^ ^]^

The aforementioned approaches either replace QM calculations with ML surrogate models or use ML algorithms as their optimization scheme, feeding all optimization targets into a single ML model. This work, in contrast, predicts only the bulk‐phase density of *n*‐octane–a single optimization target–using a ML model. One of our goals is to provide more control over the errors introduced by this substitution and, more importantly, retain the modularity of the optimization workflow. By replacing only a single target property, the trained ML model can be reused in further optimization processes, and the current optimization task can be easily expanded by other optimization objectives (i.e., target properties) without the necessity of retraining the whole ML model.

## 2 Methodology

### 2.1 The Optimization Toolkit FFLOW

**Figure 1:**
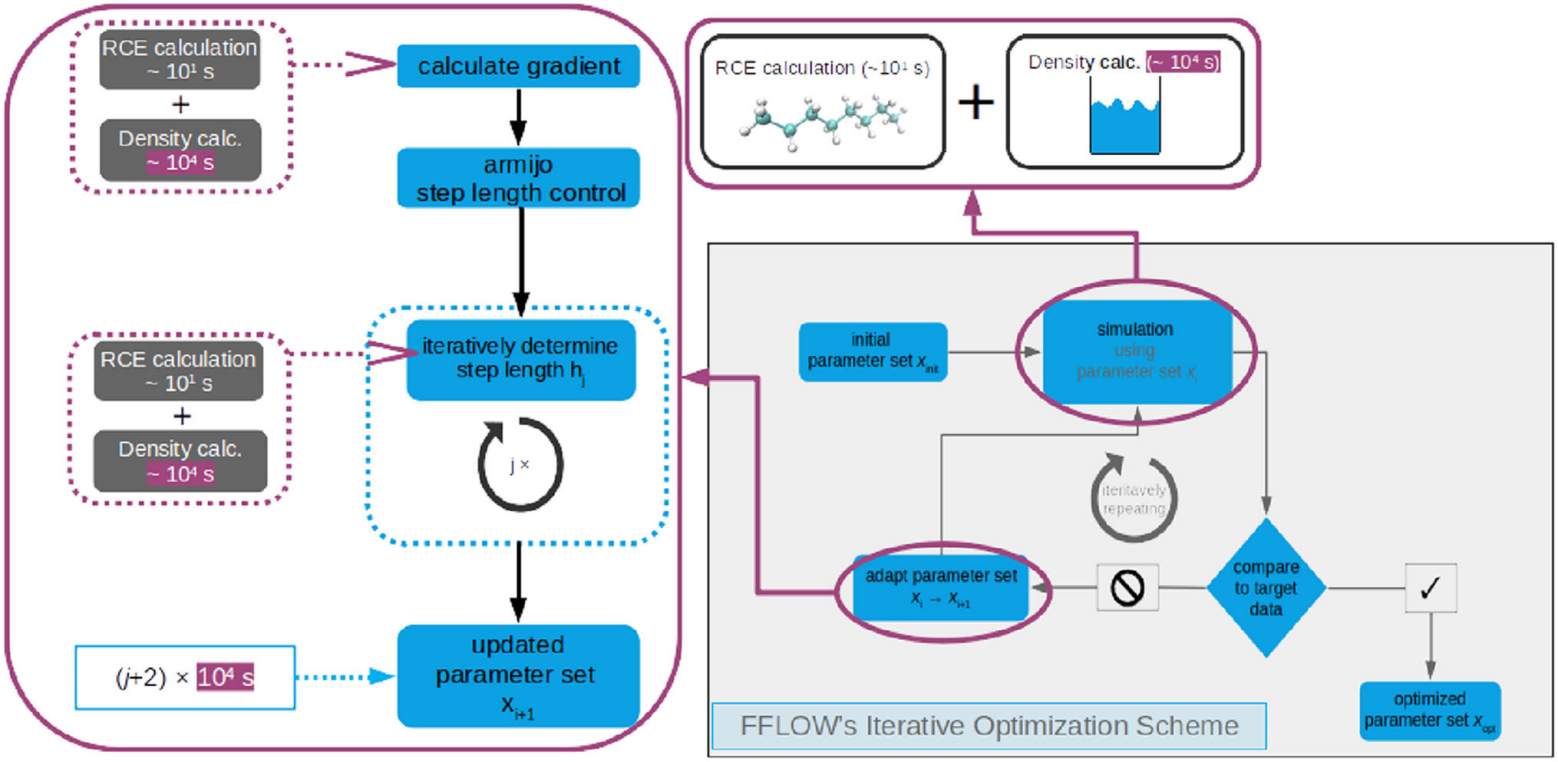
FFLOW's iterative optimization scheme: starting with an initial parameter set, the workflow compares the simulation results to the targets and adapts the FFParams based on a gradient‐descent algorithm to minimize the deviation between simulation and target. The newly proposed parameter set is then used in the next iteration, and this is repeated until the user‐defined stopping criterion is met. Ellipses highlight where FFParams are evaluated, and the connected boxes show how these evaluations are structured. Compared to the calculations of the RCE, the density MD simulations require three orders of magnitude more time to be computed.

The optimization toolbox used herein, “Force FieLd Optimization Workflow (FFLOW)”, is developed for automated multiscale FFParam optimizations. It provides a modular and iterative optimization workflow that subsequently improves a set of FFParams until a user‐defined stopping criterion is met. For every iteration, several evaluations of the FFParams, with respect to every optimization objective, are necessary. **Figure** **1** depicts the optimization workflow's scheme, highlighting the sections where the FFParams are evaluated using ellipses. In prior work, FFLOW was used to optimize LJParams w.r.t. *n*‐octane's 96 unique RCE^[^ ^28^ ^]^ (i.e., a single‐molecule, nanoscale property) and its bulk‐phase density at 293.15 K and 1 bar (i.e., a multimolecule, macroscale property).^[^ ^16^, ^17^ ^]^

During the optimization process, MM minimizations and MD simulations are performed, with the MM calculations executed within seconds to minutes, while the MD simulations require approximately three orders of magnitude longer–up to 2 h. Consequently, MD simulations consume the majority of the optimization time. Herein, we substitute the MD simulations in the optimization workflow with a data‐driven ML surrogate model to speed up the optimization process. Additionally, the process of acquiring the training data, data preprocessing, and selecting a suitable surrogate model is presented.

The optimization toolkit FFLOW, the data, and the scripts used for model training are available on GitHub (see Section “Data and Software Availability”).

### 2.2 Surrogate Model Requirements

To substitute the MD simulations, the surrogate model must accurately predict the desired optimization objective (i.e., target property) with the corresponding ambient and boundary conditions for any set of FFParams within a previously defined feasible parameter space. For this optimization, *n*‐octane's bulk‐phase density at 293.15 K and 1 bar was mapped to the LJParams (see **Table** **1**). The model's performance was judged based on the mean absolute percentage error (MAPE) and the coefficient of determination (R^2^) (i.e., the correlation) between the predictions and the target data. When preparing the model's training data, its 5‐dimensional landscape (i.e., the surface created by mapping the LJParams to the corresponding density values) was investigated to identify possible characteristics that might explain why certain surrogate models perform better. Those characteristics are, for example, multimodality or differing ruggedness across regions (i.e., alternating areas of smooth and rough surfaces), which may occur on different length scales over the entire feasible parameter space.

**Table 1:** The feasible parameter space for training data acquisition. Shown are the boundaries (i.e., minimum and maximum values) for the LJParams *σ* and *ϵ* for the data acquisition.

Parameter	Min	Max
*σ*_C_ [nm]	0.0500	0.3500
*σ*_H_ [nm]	0.0450	0.3500
*ε*_c_ [kJ mol^−1^]	0.1500	1.1500
*ε*_H_ [kJ mol^−1^]	0.0100	0.1500

### 2.3 Acquiring the Training Data

The ML models require training data to be established. This data was generated by performing (many) MD simulations using different FFParams sets to generate the property values that we want our model to predict. In our example case, by running MD simulations on a set of LJParams (*σ*_C_, *σ*_H_, *ε*_C_, *ε*_H_), we obtained octane's bulk‐phase density at 293.15 K and 1 bar.^[^ ^29^ ^]^ In case training data is already available, this step can be skipped.

#### 2.3.1 The Feasible Parameter Space

To ensure physically reasonable solutions, the user must predetermine the feasible parameter space that can be searched during the optimization. In this work, the parameter *σ* determines the distance where the long‐range attractive forces switch to the short‐range repulsive forces. Consequently, *σ* controls the equilibrium distances between interacting particles, which has a direct impact on the simulated density. The parameter *ε* corresponds to the Lennard–Jones potential's well depths and regulates how much effort (i.e., energy) is required to disturb an equilibrated system. If the well depth is too deep, the MD simulations might underestimate the influence of the temperature or pressure, and vice versa. The user‐defined feasible parameter space for this dataset is presented in Table 1. The minimal and maximal values were chosen such that they produce physically and numerically stable MD calculations.

#### 2.3.2 Sampling the Parameter Space

After defining the feasible parameter space, and if training data is not already available, the parameters were subsequently sampled. These samples and the target values resulting from MD simulations will serve as the input data for training the ML model, and can be selected using various strategies. In general, a sampling strategy needs to ensure that both the entire feasible space and the features of the data's landscape, which might occur on different length scales, are covered so that this information is available in the training data.

Herein, the following two approaches are compared: a grid‐based and a pseudo‐random Sobol sequence sampling.^[^ ^30^, ^31^ ^]^ The grid‐based approach is a commonly used simple strategy, which we anticipate will systematically miss effects at certain scale levels. The Sobol approach is a pseudo‐random strategy that should better capture the multimodality and ruggedness present in the landscape. For both approaches, two slightly different setups were used for minimizing the chance of coincidentally choosing a particularly good or bad collection of LJParams. The sampling differs in slightly varying feasible parameter spaces, resulting in different numbers of grid points (see **Table** **2**) and values for the grid‐based samples (“Grid1296” and “Grid2401”, indicating their respective sampling size) and different Sobol sequences (“Sobol1” and “Sobol2”). The 4‐dimensional Sobol sequences, with each element representing a set of LJParams, were generated using SciPy.^[^ ^32^ ^]^

**Table 2:** The four different sample sets, used as LJParams in MD simulations to acquire the training data. Shown are the minimum and maximum values for each parameter and, if applicable, the step size by which the value incrementally gets increased. Furthermore, the sample size is listed. The values in parentheses are the actual sample size of the corresponding dataset used for the model training.

	Parameter	Min	Step size	Max	#Samples (preprocessed)
Grid1296	*σ*_C_ [nm]	0.0750	: 0.0500 :	0.3250	6^4^ = 1296(1116)
	*σ*_H_ [nm]	0.0683	: 0.0455 :	0.2958	
	*ε*_c_ [kJ mol^−1^]	0.2465	: 0.1643 :	1.0680	
	*ε*_H_ [kJ mol^−1^]	0.0300	: 0.0200 :	0.1300	
Grid2401	*σ*_C_ [nm]	0.0500	: 0.0500 :	0.3500	7^4^ = 2401(1763)
	*σ*_H_ [nm]	0.0455	: 0.0455 :	0.3185	
	*ε*_c_ [kJ mol^−1^]	0.1643	: 0.1643 :	1.1501	
	*ε*_H_ [kJ mol^−1^]	0.0200	: 0.0200 :	0.1400	
Sobol1	*σ*_C_ [nm]	0.0500	–	0.3500	2^11^ = 2048(2005)
	*σ*_H_ [nm]	0.0450	–	0.3200	
	*ε*_c_ [kJ mol^−1^]	0.1500	–	1.1500	
	*ε*_H_ [kJ mol^−1^]	0.0200	–	0.1400	
Sobol2	*σ*_C_ [nm]	0.0500	–	0.3500	2^11^ = 2048(1700)
	*σ*_H_ [nm]	0.0450	–	0.3500	
	*ε*_c_ [kJ mol^−1^]	0.1500	–	1.1500	
	*ε*_H_ [kJ mol^−1^]	0.0100	–	0.1500	

#### 2.3.3 Data Preprocessing

**Figure 2:**
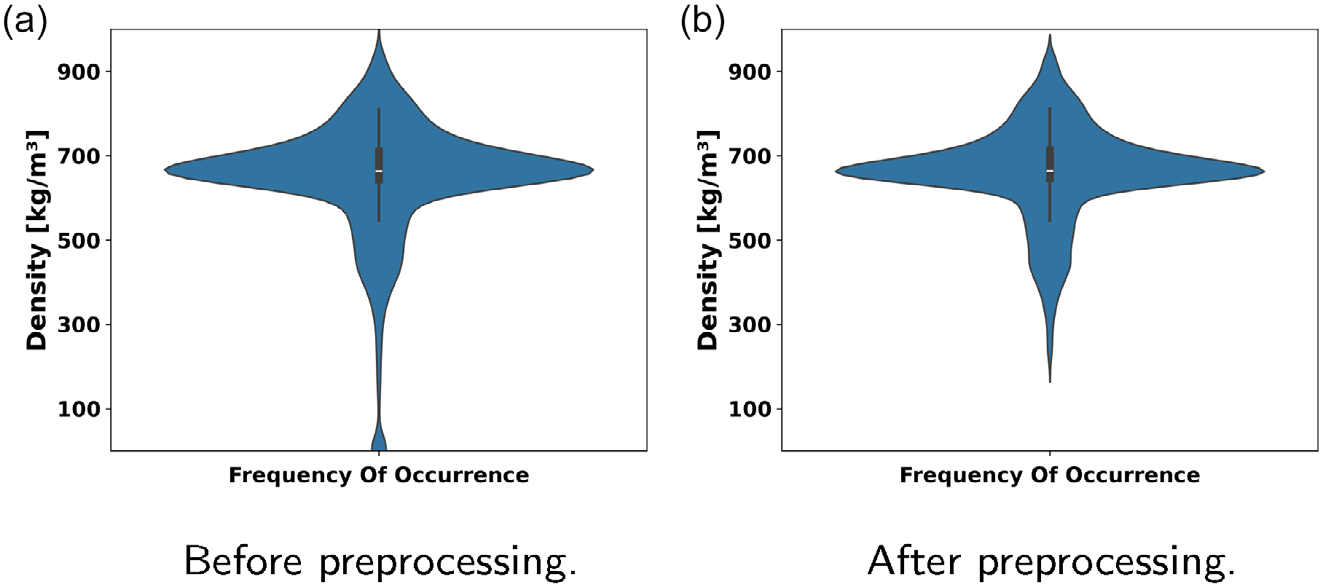
Frequency of occurrence for the density in the Sobol1 dataset a) before and b) after preprocessing.

After all MD simulations were performed, the resulting densities were merged with the corresponding LJParams to form the training data. As a preprocessing step, we removed invalid samples–for example, when a single MD simulation did not terminate successfully and the corresponding density is unrealistic or statistically unconverged. **Figure** **2** shows the frequency distribution of density value occurrences before and after preprocessing, exemplified by the Sobol1 dataset. For plots of the other datasets, refer to Figure 13–15 in the Supporting Information (SI). In Table 2, the feasible parameter space, the step sizes for the grid‐based samples, and the number of samples before and after preparing the dataset are provided.

**Figure 3:**
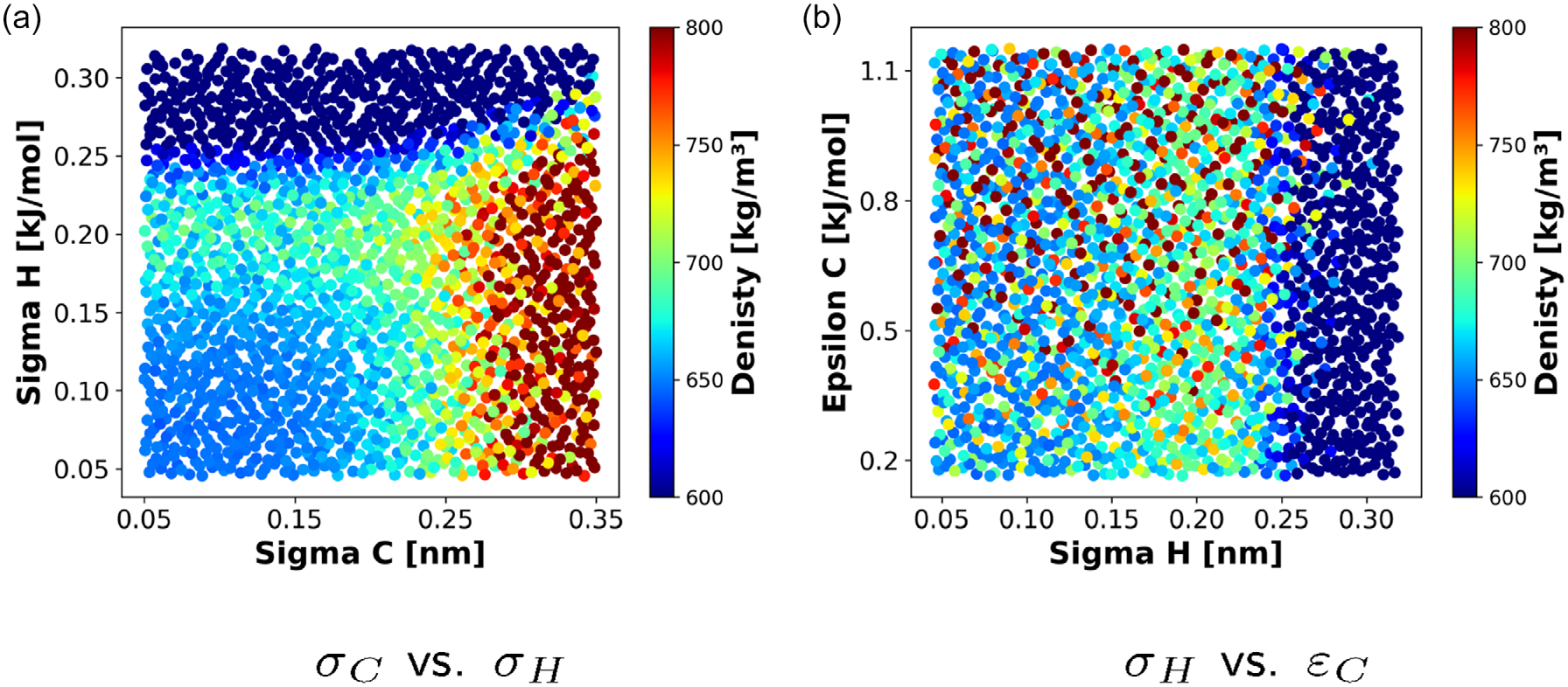
Octane's density for a selection of LJParams (σ_C_ versus σ_H_ and σ_H_ versus *ε*_C_). The target density for this optimization is 700 kg m^−3^.

To help choose a surrogate model, the sampled data is visualized to better understand the characteristics of the dataset's landscape. The density with respect to *σ*_C_ versus *σ*_H_ and *σ*_H_ versus *ε*_C_ is shown in **Figure** **3**a,b, respectively. Considering a target density of 700 kg m^−3^, the transition from “too low” to “too high” is rather smooth when examining *σ*_C_ versus *σ*_H_ coupling (Figure 3a), while there is much more noise with alternating “good” and “too low/high” densities within small changes in *σ*_H_ and *ε*_C_ (i.e., a rugh landscape; see Figure 3b). This indicates that a suitable surrogate model needs to handle different length scales and a multimodal objective landscape. Note that Figure 3 does not show all dimensions of the landscape, which can be found in Figure 12 in the Supporting Information. Furthermore, this data examination is not a formal landscape analysis, which can play an important role depending on the topic (e.g., drug design^[^ ^33^, ^34^, ^35^, ^36^ ^]^), but promotes a better understanding of why certain ML modeling methods perform better than others.

### 2.4 Training the Surrogate Models

Various surrogate models were compared to determine which ones are suitable for substituting the MD simulations. The considered approaches are a) linear regression since it is a simple and fast baseline algorithm, b) polynomial regression since it is a simple method that can model multiple optima, c) random forest regression (RFR) since it can also handle multimodality, but is more robust to overfitting than polynomial regression,^[^ ^37^ ^]^ d) Gaussian process regression (GPR) due to its capability of handling different length scales and nonlinear relations, and e) feed‐forward neural network (FNN) regression since it can model very complex and highly nonlinear correlations. There are many different neural network methods, and each excels in different applications. For example, convolutional neural networks work well for arrays as input (e.g., image recognition),^[^ ^38^ ^]^ recurrent neural networks handle sequential input structures well (e.g., speech and language recognition),^[^ ^39^ ^]^ and GNN are specialized for graph‐like inputs (e.g., predictions based on molecules;^[^ ^40^ ^]^ social media analysis^[^ ^41^ ^]^). However, for this work, a FNN was the chosen method since they are often used in problems that include complex, nonlinear regression tasks.^[^ ^42^, ^43^ ^]^ For each of the aforementioned considered modeling approaches, the following general training strategies were pursued to enable comparability and provide a robust selection strategy.

#### 2.4.1 Quantifying a Model's Performance

A model's performance was quantified by calculating the MAPE and coefficient of determination (*R* ^2^) of its predictions with respect to the dataset's target values, as implemented in Scikit‐learn.^[^ ^44^ ^]^ The data used for this performance test originates from the same sampling approach as the training data (i.e., Grid1296, etc.), and will be referred to as “in‐sample test (IST)”. Note that the testing data is split from the training data before model training, ensuring that the models have not yet “seen” the testing data.

The model's capability to accurately predict the optimization target (i.e., *n*‐octane's density) between the training data elements is an important feature since the optimization algorithm can suggest any combination of parameter values within the feasible parameter space up to machine precision. Thus and in addition to the IST, the model's MAPE and *R* ^2^ scores were calculated using the other datasets that were not used in the training. This serves as an expanded test for the model's capability to generalize beyond the samples collected through the sampling strategy and will be referred to as “out‐of‐sample test (OST)”. When, for example, a model was trained using the Grid1296 dataset, the MAPE and *R* ^2^ values for the OST were calculated using all data from the Grid2401, Sobol1, and Sobol2 as a combined testing dataset.

#### 2.4.2 Dataset Splitting Ratios

The datasets (i.e., Grid1296, Grid2401, Sobol1, and Sobol2) were split into model training and testing data with varying ratios to determine how well the models generalize to unseen data. We varied the ratios from 0.05 to 0.95 in 0.05 increments. For example, a 0.75 ratio indicates a pseudo‐random 75% split of the dataset was used for training, while the remaining 25% was used in performance tests to calculate the MAPE and *R* ^2^ score (i.e., the IST).

For every ratio, 50 individual splits were used to train the models, each having a different initial seed. The resulting MAPE and *R* ^2^ score are averaged to reduce the impact of outliers from particularly well or poorly performing models and data splits. Data splitting was performed using Scikit‐learn,^[^ ^44^ ^]^ and the initial seeds are listed in the Supporting Information.

#### 2.4.3 Model Hyperparameters

Some ML models have hyperparameters that require determination since they notably influence the models’ accuracy. For the polynomial regression, the polynomial degree *d* was varied (*d *= {1,…,10}); for the RFR, six different number of trees *t* were used (t = {10, 100, 250, 500, 750, 1000}); and for the GPR three different kernels were compared.

In total, this resulted in 3800 (i.e., 19 different data splits with 50 individual random splits for each ratio, and for each of the four datasets) different models trained for the linear regression, 38 000 models for the polynomial regression, 22 800 models for the RFR, 11 400 models for the GPR, and 3800 different FNN models.

## 3 Surrogate Model and Hyperparameter Study

### 3.1 Linear Regression

**Figure 4:**
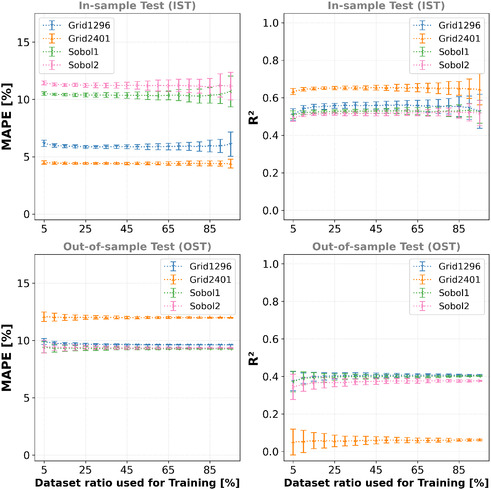
Avg. MAPE and avg. *R* ^2^ scores for the different ratios of the datasets used for the linear regression model training. For each ratio, 50 different splits (using different random seeds) are averaged. First row: IST. Second row: OST. The more elements of the datasets are used for the training, the larger is the variance of the MAPE and *R* ^2^ for the IST (i.e., overfitting). For the OST it's vice versa (i.e., underfitting when less data is used).

Linear regression is the simplest method in this comparison, and Scikit‐learn^[^ ^44^ ^]^ was used for the model trainings. The average MAPE and *R* ^2^ scores of the model trainings based on the various data split ratios for each of the four datasets are presented in **Figure** **4**.

The models trained using the grid‐based training datasets initially show better performance concerning the average MAPE and average *R* ^2^ scores than the models trained using the Sobol‐based sampling. However, when performing the OST, their performances significantly decrease (i.e., *p*‐values << 0.01 for $H_{0}\text{ : }\mu_{\text{MAPE,IST}}=\mu_{\text{MAPE,OST}}$ or 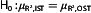). The models trained using the Sobol‐based training data are more robust toward OST and show similar MAPE and *R* ^2^ scores. The ratio used in the dataset splitting does not affect the average MAPE and average *R* ^2^ scores a lot. However, the variance for the IST increases when a larger training data amount is used (i.e., an increased chance for overfitting), while the variance for the OST is larger when less training data is used (i.e., underfitting, see Figure 4). More detailed data are presented in Figure 16 and Table 9 in the Supporting Information. Overall, the performance of the linear regression models is not sufficient, especially for the OST, since an average MAPE of ≈10% is too much, and an average *R* ^2^ of ≈0.5 indicates only a weak correlation.

### 3.2 Polynomial Regression

**Figure 5:**
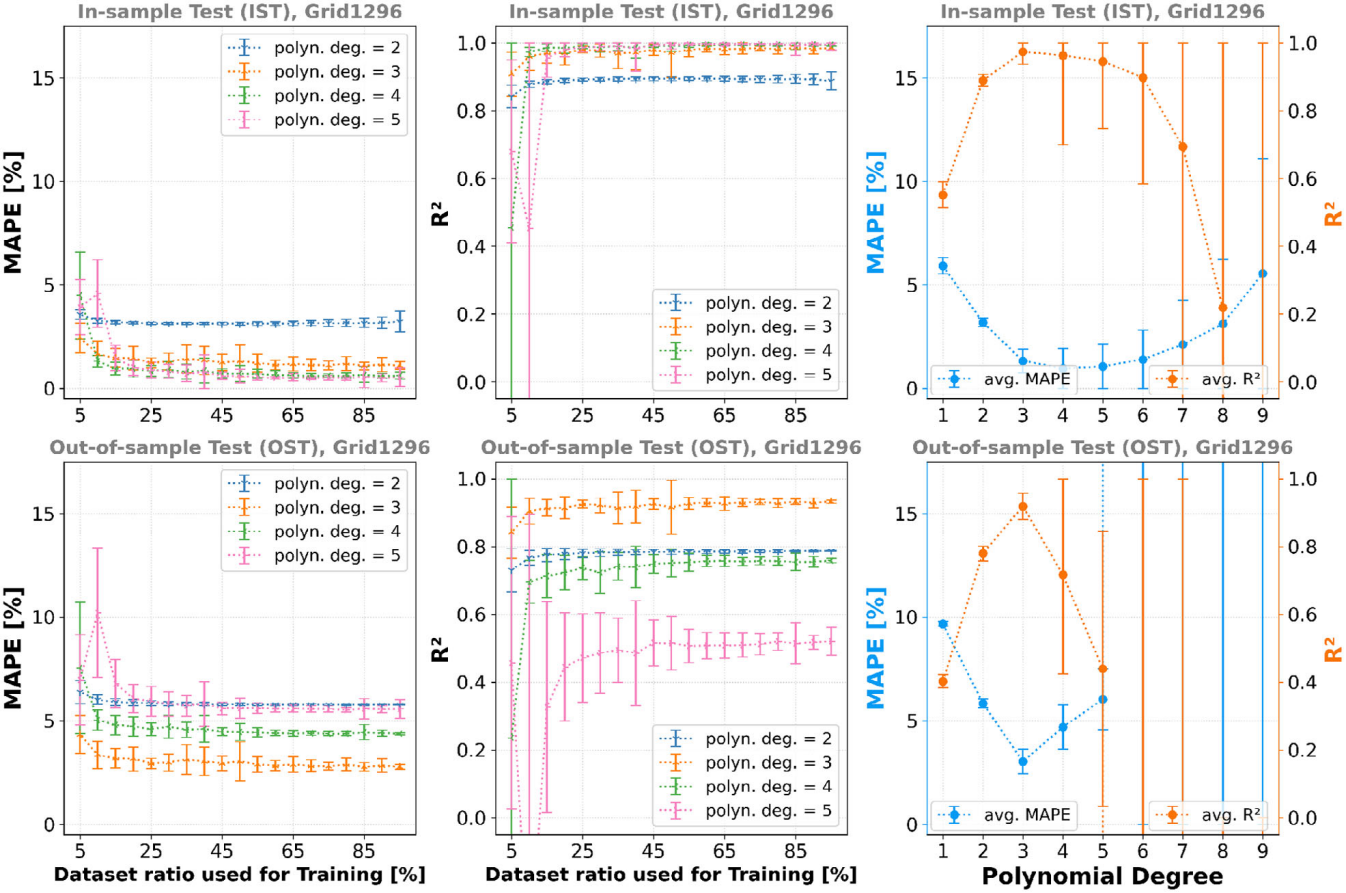
First and second column: avg. MAPE and avg. *R* ^2^ scores for the different ratios of the datasets. Plotted are the averages for the most relevant polynomial degrees *d*. For each ratio, 50 different training data splits (using different random seeds) are averaged. Third column: MAPE and *R* ^2^ score averaged over all models trained for the corresponding polynomial degree *d*. Here, an overall tendency for underfitting (d ≤ 2) and overfitting (d ≥ 4…6) can be seen. First row: IST. Second row: OST. Dataset: Grid1296.

Fitting polynomials to the training data leads to an increased capability to account for multiple optima in comparison to linear regression. Polynomial degrees *d *= {1,…,10} are compared, and Scikit‐learn^[^ ^44^ ^]^ was used to train the models. The average MAPE and average *R* ^2^ scores of the model trainings–based on the various data split ratios for a selection of polynomial degrees–are presented in **Figure** **5**, exemplified by the Grid1296 dataset. Please refer to Table 10, 11, and Figure 17–20 in the Supporting Information for the other datasets.

In the first two columns of Figure 5, the MAPE and *R* ^2^ versus the data split ratios are visualized. Although showing a good performance with low MAPE and high *R* ^2^ scores combined for the IST, when performing the OST most of the models’ performances significantly decrease (for the data presented in **Table** **3**: *p*‐values <0.01 for $H_{0}\text{ :}\;\mu_{\text{MAPE,}\;\text{IST}}=\mu_{\text{MAPE,}\;\text{OST}}$ or $H_{0}\text{ :}\;\mu_{R^{2},\;IST}=\mu_{R^{2},\;OST}$). Figure 5 additionally indicates that ≈20% of the dataset is already sufficient for the training of this model since the average MAPE and *R* ^2^ scores begin to converge. The graphs in the third column show the MAPE and *R* ^2^ score versus the polynomial degree. Depending on the dataset used for training, the optimal degree *d* is 2 ≤ *d ≤ *5.

**Table 3:** Average MAPE, average *R* ^2^, and the polynomial degree *d* for the best performing polynomial regression models for each of the training datasets, based on the IST values. To show the model's change in performance, the OST values are also presented. The complete data are presented in Table 10, 11 in the Supporting Information.

Dataset	d	Avg. MAPE	Avg. R^2^
Grid1296 (IST)	3	0.013 ± 0.006	0.974 ± 0.037
	4	0.010 ± 0.010	0.962 ± 0.263
Grid1296 (OST)	3	0.030 ± 0.006	0.919 ± 0.039
	4	0.047 ± 0.011	0.716 ± 0.292
Grid2401 (IST)	2	0.022 ± 0.002	0.845 ± 0.038
	4	0.018 ± 0.028	0.689 ± 2.031
Grid2401 (OST)	2	0.092 ± 0.003	0.370 ± 0.052
	4	0.047 ± 0.038	0.613 ± 1.397
Sobol1 (IST)	4	0.018 ± 0.007	0.979 ± 0.029
	5	0.014 ± 0.013	0.970 ± 0.101
Sobol1 (OST)	4	0.025 ± 0.008	0.905 ± 0.071
	5	0.022 ± 0.015	0.883 ± 0.280
Sobol2 (IST)	3	0.029 ± 0.015	0.945 ± 0.202
	5	0.021 ± 0.025	0.908 ± 0.572
Sobol2 (OST)	3	0.027 ± 0.014	0.892 ± 0.307
	5	0.022 ± 0.023	0.812 ± 1.219

### 3.3 RFR

**Figure 6:**
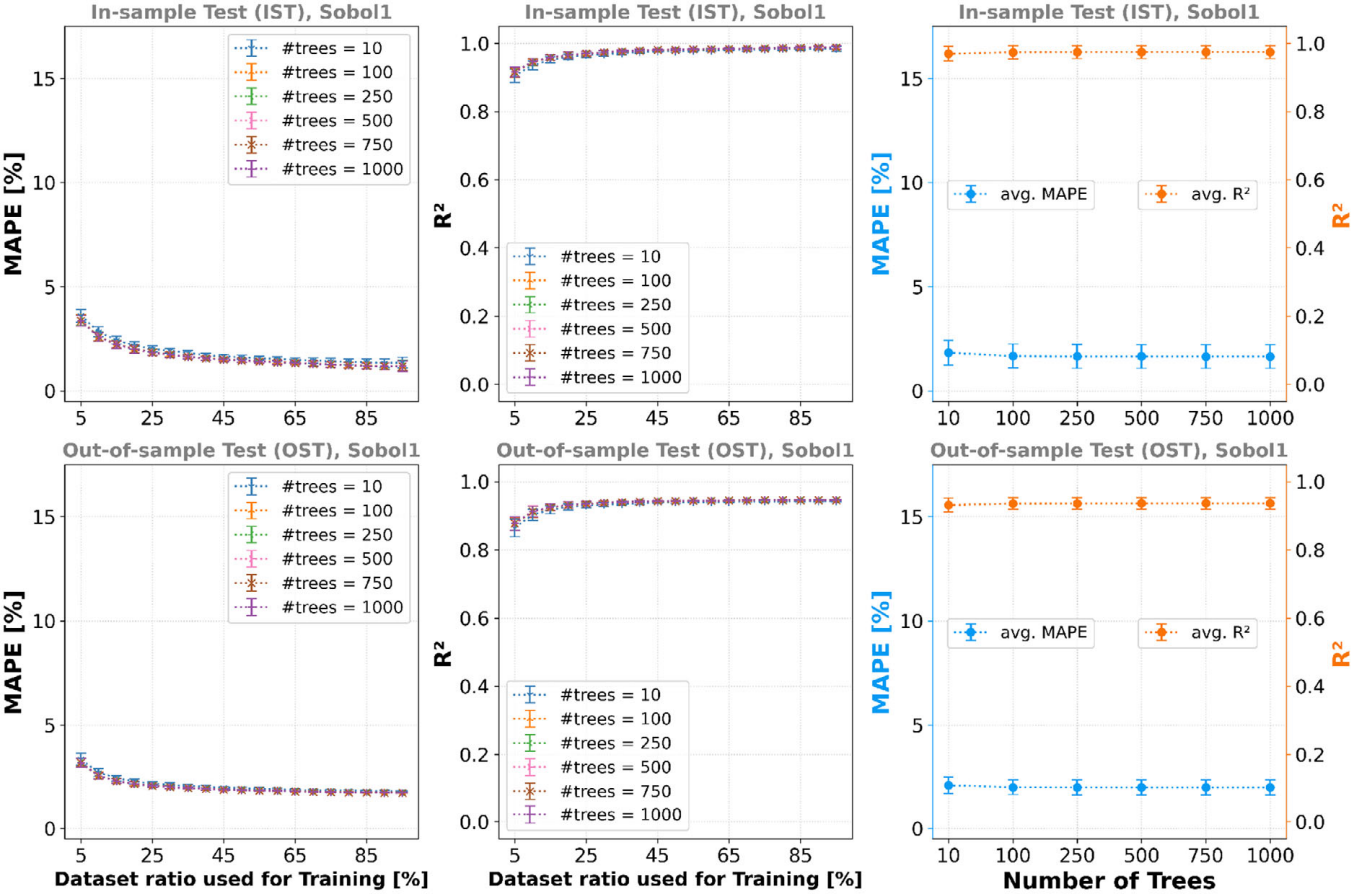
First and second column: avg. MAPE and avg. *R* ^2^ scores for different the ratios of the dataset and the different numbers of trees used for the model training. For each ratio, 50 different training data splits (using different random seeds) are averaged. Third column: MAPE and *R* ^2^ score averaged over all models trained for the corresponding number of trees. Here, no under‐ or overfitting can be seen. First row: IST. Second row: OST. Dataset: Sobol1.

RFR is also capable of modeling nonlinear correlations and multimodality in the training datasets. Compared to polynomial regression, it is less prone to overfitting.^[^ ^37^ ^]^ For each of the aforementioned training setup combinations, different numbers of trees t={10, 100, 250, 500, 750, 1 000} are compared, and Scikit‐learn^[^ ^44^ ^]^ was used to carry out the model trainings. The average MAPE and average *R* ^2^ scores of the model trainings based on the various data split ratios for the different numbers of trees are shown in **Figure** **6**.

Figure 6 indicates that increasing the number of trees beyond t > 100 does not significantly affect the MAPE and *R* ^2^ scores. Significance tests for the null‐hypotheses $H_{0,\;MAPE}\text{ :}\;\mu_{MAPE,\;t=100}=\mu_{MAPE,\;t\geq 250}$ and $H_{0,\;R^{2}}\text{ :}\;\mu_{R^{2},\;t=100}=\mu_{R^{2},\;t\geq 250}$ (i.e., that the mean MAPE and *R* ^2^ scores do not differ for 100 trees and more than 250 trees) result in *p*‐values ≥0.45, which means that $H_{0,\;MAPE}$ and $H_{0,\;R^{2}}$ cannot be rejected. However, for $H_{0,\;MAPE}\text{ :}\;\mu_{MAPE,\;t=10}=\mu_{MAPE,\;t\geq 100}$, the significance tests result in *p*‐values _*MAPE*_>0.80 and for $H_{0,\;R^{2}}\text{ :}\;\mu_{R^{2},\;t=10}=\mu_{R^{2},\;t\geq 100}$, they result in *p*‐values *R* ^2^ = {0.30, 0.04, 0.06, 0.04} for Grid1296, Grid2401, Sobol1, and Sobol2, respectively. Thus, taking both MAPE and *R* ^2^ into account, *t* = 100 should be preferred over *t* = 10. Compared to polynomial regression, the RFR did not show overfitting when increasing the number of trees to up to *t* = 1000. Note that Figure 6 shows the data for models trained using the Sobol1 dataset. The models trained with the other datasets show a similar trends, except that the models trained on the Grid1296 and Grid2401 datasets perform worse for the OST than the models trained on the Sobol1 and Sobol2 datasets (see Table 12, 13, and Figure 21–24 in the Supporting Information).

In **Table** **4**, the best RFR models for every training dataset are outlined. Note that for the trainings using the Sobol1 and Sobol2 datasets, the results for the number of trees of *t* = 100 are equally good as using *t* = 750, considering the variance. Thus, increasing the number of trees to 750 for this training is not meaningful, since it mostly increases the required time to train the models.

**Table 4:** Average MAPE, average *R* ^2^, and the number of trees #*t* for the best performing RFR models for each of the training datasets, based on the IST values. To show the model's change in performance, the OST values are also presented. The complete data is presented in Table 12, 13 in the Supporting Information.

Dataset	#t	Avg. MAPE	Avg. *R* ^2^
Grid1296 (IST)	100	0.009 ± 0.005	0.976 ± 0.039
Grid1296 (OST)	100	0.047 ± 0.005	0.825 ± 0.051
Grid2401 (IST)	100	0.012 ± 0.003	0.857 ± 0.044
Grid2401 (OST)	100	0.094 ± 0.004	0.322 ± 0.047
Sobol1 (IST)	100	0.017 ± 0.006	0.974 ± 0.019
Sobol1 (OST)	100	0.020 ± 0.004	0.936 ± 0.018
Sobol2 (IST)	100	0.022 ± 0.007	0.961 ± 0.023
Sobol2 (OST)	100	0.019 ± 0.004	0.946 ± 0.023

### 3.4 GPR

GPR is a well‐defined statistical method for regression tasks. Its advantages are that they not only provide the mean value of a prediction but also the resulting uncertainty. It is composed of joint Gaussians that are fitted to the training data, enabling it to model very complex nonlinear relationships.^[^ ^42^ ^]^ However, the kernel selection is crucial and determines the modeling of the correlation between training points, and how strongly they correlate with the prediction at a point of evaluation. A kernel can be, for example, a radial basis function (RBF; i.e., a local Gaussian) or a periodic function, which means that a training sample's function value affects evaluation points periodically.

**Figure 7:**
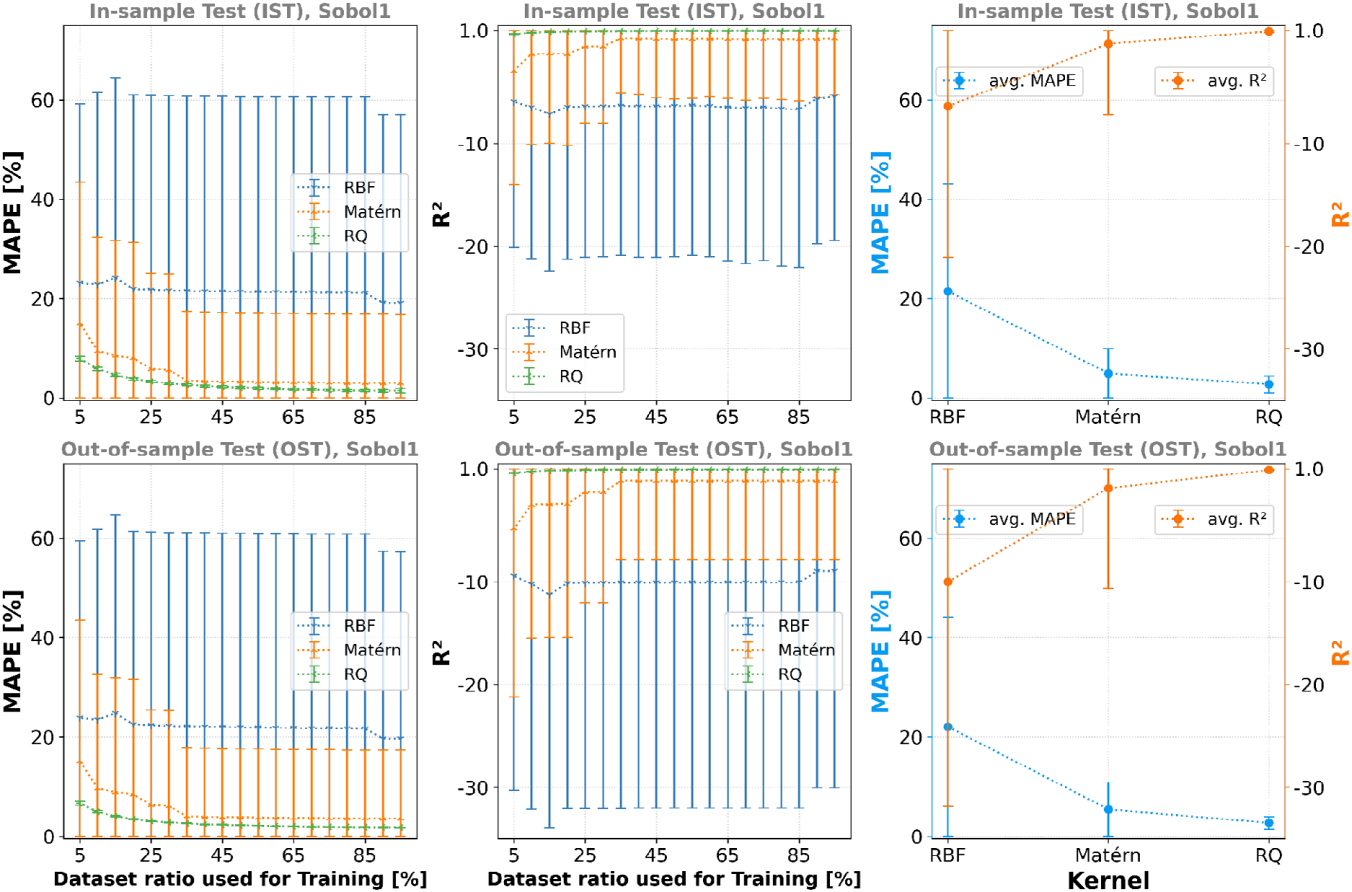
First and second column: avg. MAPE and avg. *R* ^2^ scores for different the ratios of the dataset and the different kernels used for the model training. For each ratio, 50 different training data splits (using different random seeds) are averaged. The average performance of the RBF and Matérn is worse than for the RQ kernel. However, their variance is very large, and well‐performing models exist. Third column: MAPE and *R* ^2^ score averaged over all models trained for the corresponding kernel. First row: IST. Second row: OST. Dataset: Sobol1.

Here, three different kernels are compared, and Scikit‐learn^[^ ^44^ ^]^ was used to train the GPR models. The kernels are the RBF^[^ ^45^ ^]^ kernel, which is one of the most commonly used kernels; the Matérn^[^ ^42^, ^46^ ^]^ kernel, which is a generalization of the RBF kernel with an additional parameter to control the smoothness; and the rational quadratic (RQ)^[^ ^42^, ^47^ ^]^ kernel, which is also a variation of the RBF kernel–allowing a mixture of different characteristic length scales. The smoothness parameter or characteristic length scale is kernel hyperparameters that need to be adjusted during training to capture the actual local correlation in the training data. **Figure** **7** shows the model trainings’ average MAPE and average $R^{2}$ scores based on the Sobol1 dataset and various data split ratios for the different kernels (see Table 14, 15 in the Supporting Information for the values regarding the other training datasets).

On average, the Matérn and RQ kernels yielded better results than the RBF kernel. However, the variance for the RBF kernel is large, and models with a low MAPE and high $R^{2}$ score do exist. It is not surprising that the RQ kernel models perform well with a small variance, because the mixture of the characteristic length scales enables fitting of the aforementioned multimodal dataset landscape well, including its effects on different length scales. It also shows that ≈30% of the data is sufficient for this training using the RQ or Matérn kernel. In **Table** **5**, the average MAPE and average *R* ^2^ for the RQ kernel (i.e., the best performing kernel) are presented. The OST shows that the models trained with the Sobol1 and Sobol2 datasets are more robust toward OST, while the MAPE and *R* ^2^ scores for the models trained using the Grid1296 and Grid2401 datasets noticeably worsen (see Tables 14 and 15 in the Supporting Information for values for the other kernels and Figure 28 in the Supporting Information for a graphical overview of all models). Note that the graphs presented in Figure 7 are based on the trainings using the Sobol1 dataset. The graphs showing the models trained using the other datasets are presented in Figure 25–27 in the Supporting Information.

**Table 5:** Average MAPE and average *R* ^2^ for the best performing GPR models (i.e., using the RQ kernel) for each of the training datasets, based on the IST values. To show the model's change in performance, the OST values are also presented. The complete data is presented in Table S14,S15 in the Supporting Information.

Dataset	Avg. MAPE	Avg. *R* ^2^
Grid1296 (IST)	0.023 ± 0.011	0.863 ± 0.117
Grid1296 (OST)	0.068 ± 0.010	0.666 ± 0.110
Grid2401 (IST)	0.018 ± 0.005	0.842 ± 0.056
Grid2401 (OST)	0.098 ± 0.004	0.255 ± 0.038
Sobol1 (IST)	0.028 ± 0.017	0.932 ± 0.081
Sobol1 (OST)	0.027 ± 0.013	0.895 ± 0.089
Sobol2 (IST)	0.036 ± 0.019	0.907 ± 0.094
Sobol2 (OST)	0.027 ± 0.013	0.891 ± 0.104

### 3.5 Neural Network Regression

**Figure 8:**
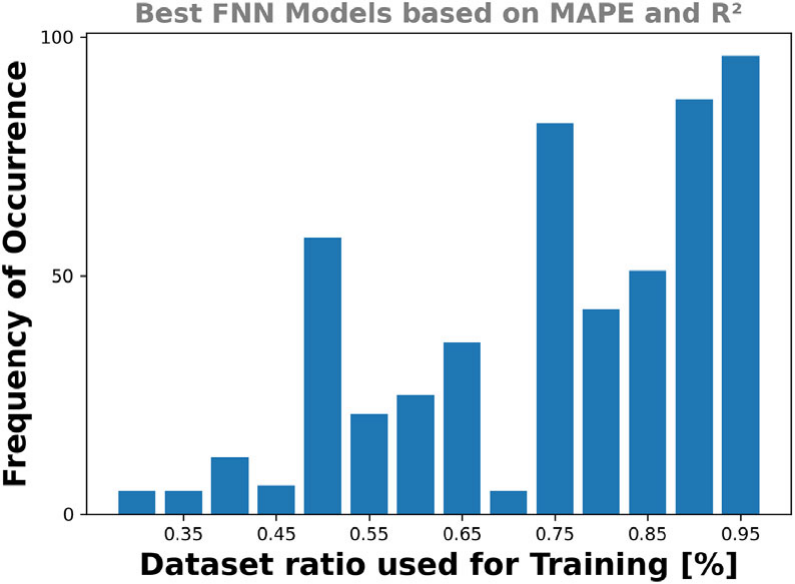
Frequency of occurrence for the dataset split ratios (i.e., how much of a dataset is used for the model's training) of the best FNN models. Using more data for the model training correlates with a higher probability to obtain a well‐performing model.

The herein trained neural networks (NNs) are feed‐forward neural networks (FNNs) that are suitable for pattern recognition and regression tasks for low‐dimensional input data.^[^ ^42^, ^43^ ^]^ Their structure – nodes that are connected with nonlinear activation functions – makes FNN a rather complex but very versatile regression method. For creating and training the FNN models, the PyTorch framework^[^ ^48^ ^]^ and the FNN architecture and parameters (see Table 16 in the Supporting Information) were used. The histogram in **Figure** **8** is based on the best performing FNN models (w.r.t. the MAPE and *R* ^2^). It shows that most of the well‐performing models are trained using a higher ratio for the dataset splitting. The aforementioned complexity of NN is reflected in the trained model's performances, showing large variances in the MAPE and *R* ^2^ scores (see Figure 29 in the Supporting Information). An overview of the MAPE and *R* ^2^ scores across all models is presented in Figure 30 in the Supporting Information.

The best FNN models (see **Table** **6**) show a combination of a low MAPE and a high *R* ^2^ score (i.e., close to 1), and their performance, especially concerning the OST, is better compared to the linear regression, polynomial regression, RFR, and GPR models.

**Table 6:** The 12 best FNN models regarding the lowest MAPE (named “M‐”) and highest *R* ^2^ score (named “R‐”) that are used in the SMAOpt. Duplicate models are indicated by listing their corresponding model names (“corresp. to”) in gray. All models are trained using the Sobol1 dataset.

Name	MAPE	$R^{2}$	Corresp. to
M‐1	0.00884	0.99237	R‐8
M‐2	0.00897	0.99340	R‐2
M‐3	0.00949	0.99258	R‐6
M‐4	0.00953	0.99398	R‐1
M‐5	0.00953	0.99326	R‐3
M‐6	0.00964	0.98962	–
M‐7	0.01004	0.99267	R‐4
M‐8	0.01005	0.99191	R‐10
M‐9	0.01016	0.99089	–
M‐10	0.01034	0.99062	–
M‐11	0.01041	0.99005	–
M‐12	0.01044	0.98991	–
R‐1	0.00953	0.99398	*M*‐4
R‐2	0.00897	0.99340	*M*‐2
R‐3	0.00953	0.99326	*M*‐5
R‐4	0.01004	0.99267	*M*‐7
R‐5	0.01056	0.99263	–
R‐6	0.00949	0.99258	*M*‐3
R‐7	0.01057	0.99238	–
R‐8	0.00884	0.99237	*M*‐1
R‐9	0.01109	0.99231	–
R‐10	0.01005	0.99191	*M*‐8
R‐11	0.01119	0.99146	–
R‐12	0.01045	0.99101	–

### 3.6 Model Selection

**Figure 9:**
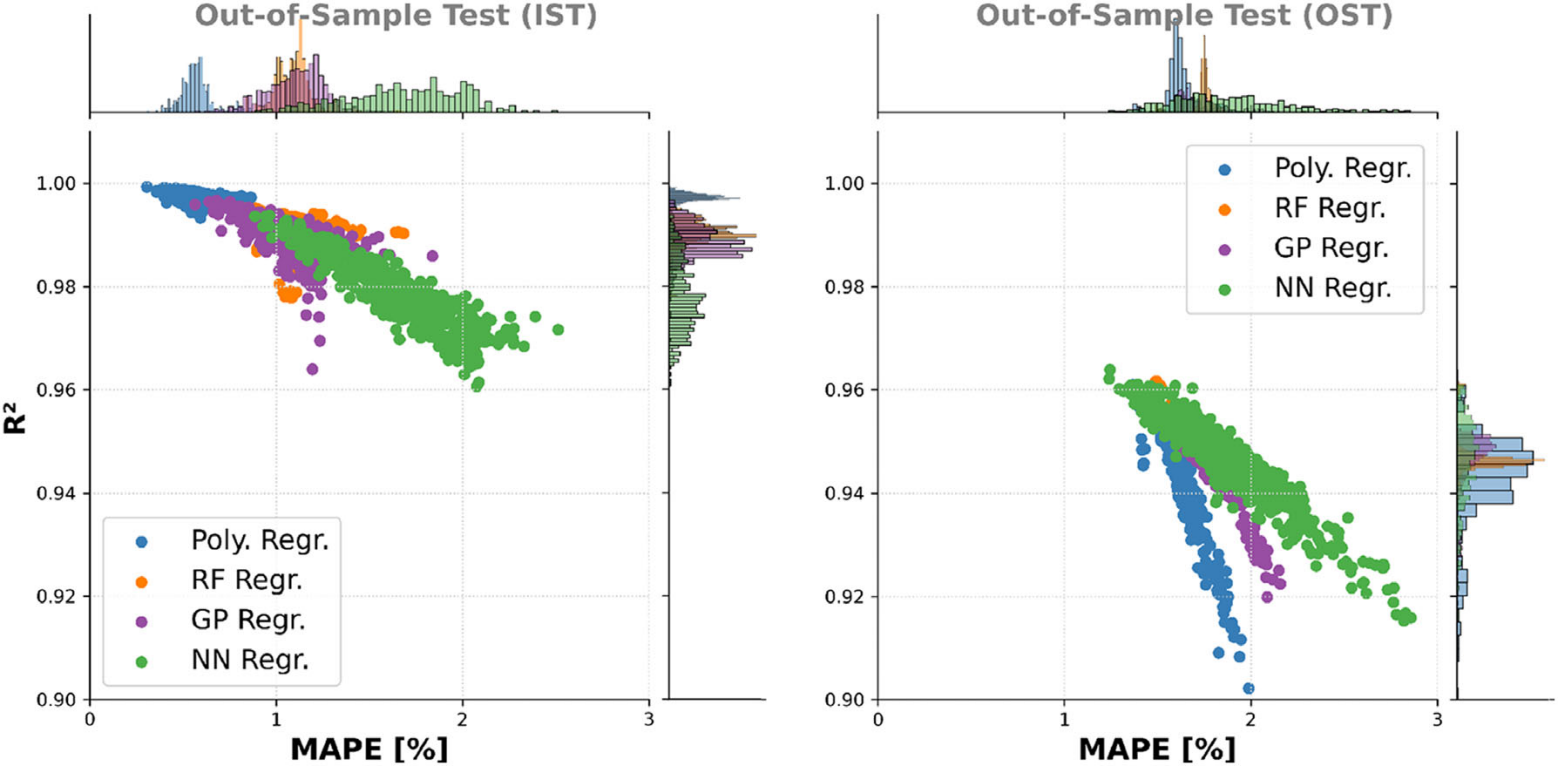
Overview of the models with the 500 best MAPE and best 500 *R* ^2^ scores (without duplicates) for the different training methods. The left graph shows the MAPE versus *R* ^2^ score regarding the IST, while the right graph shows these values w.r.t. to the OST. Due to axis scaling, the linear regression models are not shown (see Figure 31 in the Supporting Information for the inclusion of the linear regression models). The histograms above and on the right show the distribution of the MAPE and *R* ^2^, respectively.

Using linear regression, the multimodal target landscape, with its features on different length scales, cannot be modeled well. Polynomial regression can handle the multimodality, but is prone to overfitting, which can be prevented by using RFR or GPR. The GPR models’ performance strongly depends on the used kernel. However, all models were outperformed by the best FNN models in terms of combined MAPE and *R* ^2^ scores (see **Figure** **9**) when they are used outside of the originally sampled distribution (i.e., OST), which is required for the substitution of MD simulations. Note that even though the overall best surrogate models are the FNN models, on average, they do not perform significantly better than the RFR and GPR models (i.e., *p*‐values ≪ 0.01 for $H_{0}{\text{:μ}}_{\text{MAPE,}\;\text{NN}}{\text{=μ}}_{\text{MAPE,}\;\text{RFR}}$; $H_{0}{\text{:μ}}_{\text{MAPE,}\;\text{NN}}{\text{=μ}}_{\text{MAPE,}\;\text{GRP}}$ ; $H_{0}\text{ :}\;\mu_{R^{2},\;NN}=\mu_{R^{2},\;RFR}$ and $H_{0}\text{ :}\;\mu_{R^{2},\;NN}=\mu_{R^{2},\;RFR}$). Consequently, due to their computationally required resources, shallow learning models hold a cost‐to‐performance advantage.

For the linear regression, the amount of training data has a small impact on the model's performance (see Figure 4), while for the polynomial RFR and GPR ≈25% to 35% of the datasets (≈400…700 samples) showed convergence for the MAPE and *R* ^2^ scores (see Figure 5, 6, 7). The FNN models showed their best performances when 85% to 95% of the datasets were used for the training (see Table 17 in the Supporting Information), indicating that the sampling size is sufficient.

**Figure 10:**
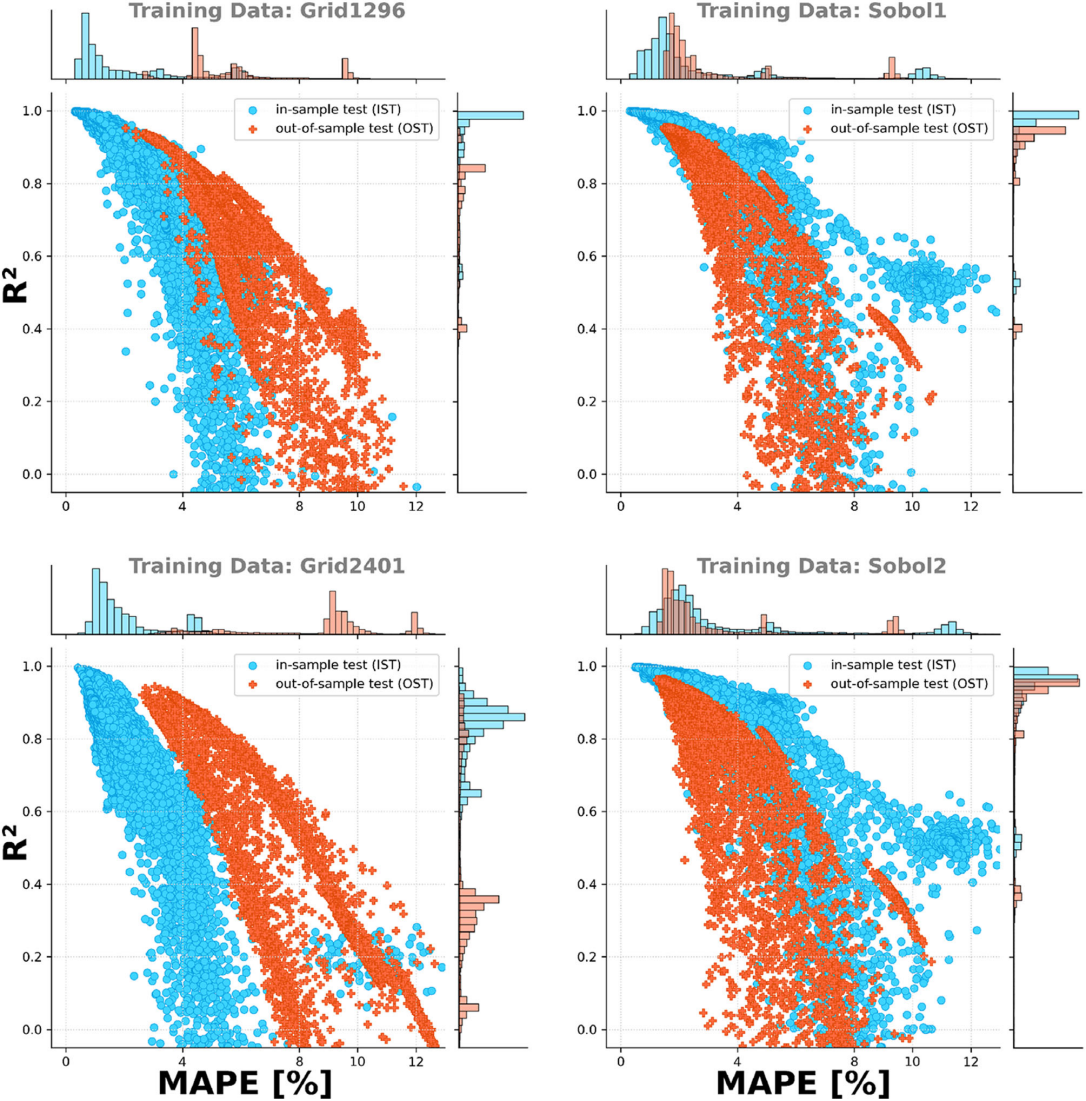
*R* ^2^ scores plotted against the MAPE of all models trained by linear regression, polynomial regression, RFR, GPR, and FNN combined and split by the dataset used for the training. Comparing the OST (orange) to the IST (blue), models trained using the Grid1296 and Grid2401 (first column) perform noticeably worse than the models trained based on the Sobol1 and Sobol2 datasets (second column). The histograms above and on the right show the distribution of the MAPE and *R* ^2^, respectively.

In summary, most of the models trained using the Grid1296 and Grid2401 datasets showed better MAPE and *R* ^2^ scores than the models trained on the Sobol1 and Sobol2 datasets, but only for the IST. When performing the OST, models trained using the Sobol1 and Sobol2 datasets showed a better performance (see **Figure** **10**). When using an equidistant, grid‐like sampling strategy, instead of a pseudo‐random sequence, it is more likely to miss features of the target optimization landscape that occur at different scale levels.

## 4 SMAOpts

For the SMAOpts, MD simulations (i.e., the “density calc.” step in Figure 1) were substituted by selected FNN models. Specifically, we utilized the 12 FNN models with the lowest MAPE and the 12 with the highest *R* ^2^ score (Table 6). This resulted in 17 different optimizations, since 7 of the models with the highest *R* ^2^ score are among the 12 models with the lowest MAPE. For the training of those models, the Sobol1 dataset was used (see Table 17 in the Supporting Information). In other words, for this multiscale optimization of octane's LJParams, every evaluation of the FFParams with regards to the density is a prediction by a ML model instead of a time‐consuming MD simulation, while the optimization settings are identical to our previous study.^[^ ^17^ ^]^

Two of the performed SMAOpts (now referred to as SMAOpts‐1 and SMAOpts‐2) lead to optimized LJParams that can reproduce the density and RCE with similar errors compared to the previous optimization^[^ ^17^ ^]^ (referred to as “PrevOpt”). The SMAOpt‐1 and SMAOpt‐2 values of the LJParams are similar to the PrevOpt parameters, but not identical – especially *ε*_C_ and *ε*_H_ differ slightly (see **Table** **7**). However, Figure 3 shows that there is a wide range of suitable *ε*_C_ and *ε*_H_, while the range for *σ*_C_ and *σ*_H_ is more constricted.

**Table 7:** SMAOpt's resulting optimized LJParams (*σ*_C_, *σ*_H_, *ε*_C_, *ε*_H_), the corresponding predicted density (*ρ*_pred_), and the calculated density (*ρ*_sim_) using MD simulations with the optimized LJParams. Additionally, their deviation from the experimental target density of 700 kg m^−3^^[^ ^29^ ^]^ (err_dens_) and the average deviation from the target RCE^[^ ^28^ ^]^ (err_RCE_) are shown. For comparison, the results of the previous optimization (PrevOpt^[^ ^17^ ^]^), which does not utilize surrogate models, are also shown.

	PrevOpt	SMAOpt‐1	SMAOpt‐1
*σ*_C_ a)	0.3286	0.3295	0.3280
*σ*_H_ a)	0.2606	0.2624	0.2653
*ε*_C_ b)	0.6730	0.5969	0.5977
*ε*_H_ b)	0.1194	0.1010	0.1020
*ρ*_pred_ c)	–	709.2	690.6
*ρ*_sim_ c)	707.0 ± 4.7	707.4 ± 1.0	693.2 ± 1.1
err_dens_ d)	1.0 ± 0.7	1.1 ± 0.1	−1.0 ± 0.2
err_RCE_ d)	9.75	10.82	11.97

a) [nm].

b) [kJ mol^−1^].

c) [kg m^−3^].

d) [%]

**Figure 11:**
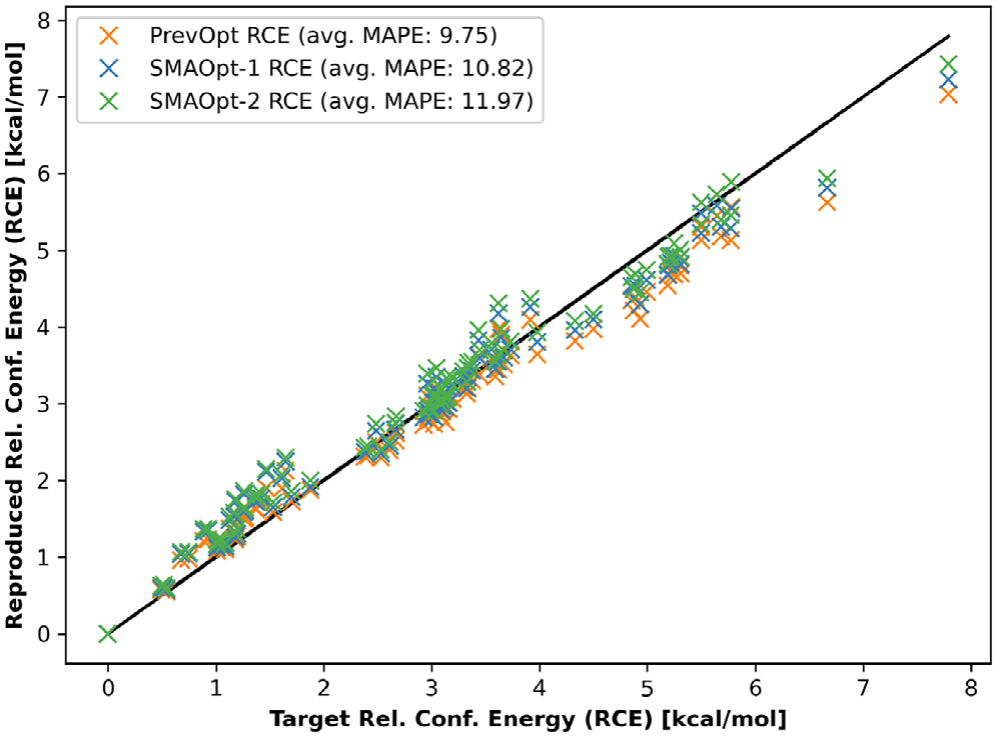
RCE calculated using LJParams optimized with the SMAOpt workflows and the previously optimized LJParams (PrevOpt RCE). The RCE are very similar, and many points are overlapping.

The target RCE versus their computed values, using the SMAOpt‐1 and SMAOpt‐2 LJParams, are plotted in **Figure** **11**. For comparison and underlining their similarity, the PrevOpt RCE^[^ ^17^ ^]^ is included.

Table 7 and Figure 11 show that the density and RCE reproduced using the herein optimized LJParams are comparable to the previously achieved optimization results. However, the necessary time to perform the optimization(s) is noticeably decreased when focusing only on the required time to terminate the optimization run. **Table** **8** shows the runtimes using the regular and the SMAOpt workflows. The required time for the optimization is reduced from just over three days to ≈ 4 hours (i.e., a factor of ≈20).

**Table 8:** Runtimes for the optimizations to terminate. Note that the same hardware was used to perform those optimization runs.

Optimization run	Total time [h]
PrevOpt	73.50
SMAOpt‐1	4.00
SMAOpt‐2	2.25

Note that different optimization approaches may need different iteration amounts to converge, and for every iteration, a different amount of FFParam evaluations is required to determine how much the current parameter set needs to be changed (i.e., the step length control). For example, PrevOpt and SMAOpt‐1 required 7 iterations, while SMAOpt‐2 required 5. How many iterations are necessary is determined by the path taken through the optimization landscape. By using a surrogate model, the optimization paths are slightly altered since the landscape's shape is dependent on how the LJParams are mapped to the optimization objective (i.e., the density). This mapping varies slightly for each surrogate model, resulting in slightly divergent optimization paths that can terminate after different numbers of iterations.

## 5 Discussion and Conclusion

The previously published multiscale optimization of the carbon and hydrogen LJParams resulted in a parameter set that reproduces *n*‐octane's bulk‐phase density and RCE with errors of 1.0% ± 0.67% and 9.75%, respectively.^[^ ^17^ ^]^ That optimization run required 73.5 h to converge. The work herein significantly reduces the required time for multiscale FFParam optimization, achieving an approximate 20‐fold run‐time reduction. This reduction is achieved by substituting the time‐consuming MD simulations with a faster ML surrogate model, and this enhanced workflow is called SMAOpt. The resulting LJParams (Table 7) are very similar to the previously obtained optimized parameters and results – the SMAOpt's errors for the density reproduction are 1.1% ± 0.1% and −1.0% ± 0.2%, while for the RCE reproduction, the errors are 10.82% and 11.97%. Thus, very similar results are achieved while requiring noticeably less time for the optimization (i.e., 4 h and 2.25 h; Table 7 and 8 and Figure 11). Given the strong similarity between the resulting LJParams and our previously published values, the transferability study published in Ref. [17] was not repeated here, as the parameters’ behavior is expected to be similar.

The ML surrogate model(s) that showed the best performance, with respect to the requirements, are trained using a FNN. Due to the training data's multimodal landscape, which exhibits effects on different length scales, linear regression is not a suitable training method. Polynomial regression can map this landscape better, but is prone to overfitting. More robust against overfitting are RFR and GPR, however, a fitting kernel for GPR is crucial and needs to suit the training data. Here, the RQ kernel, with its parameter for mixed length scales, shows good performance. However, RFR and GPR are outperformed by FNNs, especially when the OST is performed. Low errors for the prediction based on out‐of‐sample inputs are important for the SMAOpt. A crucial element for the ML trainings is the training data itself, how it is composed, and how the sampling is conducted. To ensure that characteristics of the optimization objective's landscape are not missed, we suggest that a (pseudo‐)random sampling strategy is chosen over an equidistant, grid‐like sampling, when possible. Using a ML surrogate model turns a finite set of elements (i.e., the training data) into a continuous surface. This continuity is a crucial property and is required for an optimization workflow that suggests continuous optimized solutions (i.e., any combination of FFParams within the feasible parameter space).

By substituting, only the MD simulations and not including the RCE in the ML model, the modularity of FFLOW's multiscale optimization workflow is retained. This allows changes in the optimization targets, especially adding further properties or substances, without the necessity to repeat the entire ML training process when all optimization targets are combined in a single ML model. Additionally, the isolated FNN model mapping the LJParams to *n*‐octane's density can be reused in other optimization approaches.

Decreasing the required time for FFParam optimizations makes the exploration of parameter space more feasible, but this comes at the cost of investing time and resources before the optimization itself. If there is no training data already available, it needs to be generated, which might not always be possible or affordable. Additionally, a suitable ML method must be chosen that fits the optimization objective's characteristics and meets the workflow's requirements (e.g., the capability of reproducing out‐of‐sample inputs well). Finally, there is the training itself, which can be resource‐intensive. Note that all models trained herein required less than 5 min per training, which is inconsequential compared to the time required for the multiple MD simulations. To ensure that the ML model is trained properly, it should be repeated using different initial seeds, and we suggest performing a few optimization runs using a selection from the best models because this helps to estimate the error range and identify potential outliers. Considering the necessary time for the aforementioned preparations, the overall time saved to perform the entire optimization is less than Table 8's indicated factor of ≈20. Nevertheless, while the SMAOpt workflow has an initial resource investment, it can also increase the knowledge about the optimization objective(s) (e.g., about the characteristics of the hypersurface mapping the LJParams to the density) and lead to faster optimization runs. Thus, for every optimization task, the user should assess if it is worth investing the time and resources in a) acquiring and preparing the training data, b) exploring and selecting a suitable ML model based on the optimization objective's characteristics, and c) training those models.

Beyond reducing the actual optimization time and providing more insights into the optimization objective's characteristics, the SMAOpt workflow offers further advantages. These include the reusability of the raw data and the trained ML models. Moreover, once trained, the ML surrogate model demands fewer computational resources than MD simulations, which reduces the required hardware specifications (e.g., CPU power). However, note that the initial training of the ML model requires substantially more computational power than its subsequent evaluations, which may not be feasible on all hardware configurations.

## 6 Outlook

In future work, the next meaningful steps include the usage of an optimization algorithm that provides multiple optimized parameter sets or adding additional optimization objectives. Given the multimodal nature of the landscape, as shown in Figure 3, an algorithm capable of identifying several equally well‐optimized FFParams is highly desirable. This approach allows the user to further explore multiple solutions (e.g., for transferability) and can lead to a deeper understanding of the investigated system. By adding more properties, a temperature range, or similar substances to the optimization objectives, the number of minima in the optimization landscape could decrease and lead to parameter sets with increased transferability. Furthermore, a full formal landscape analysis offers several key advantages. It can provide a deeper understanding of the entire optimization problem and enable a more generalized analysis of suitable surrogate model types and parameterizations. This approach could also significantly reduce the investment required in the model selection step. Finally, an improved objective landscape understanding will facilitate the choice of the most suitable optimization strategy.

After substituting the MD simulations with ML surrogate models, the MM minimizations become the most time‐consuming element of the optimization workflow. Thus, an additional refinement strategy would be to also surrogate‐replace those calculations, further decreasing the required optimization time.

## Conflict of Interest

The authors declare no conflict of interest.

## Author Contributions

The author contributions follow the CRediT taxonomy (https://casrai.org/credit). **Robin Strickstrock:** conceptualization; data curation; formal analysis; investigation; methodology; project administration; software; validation; visualization; writing—original draft and writing—review & editing. **Alexander Hagg**: formal analysis; and writing—review & editing. **Dirk Reith**: conceptualization; funding acquisition; resources; supervision; visualization; and writing—review & editing. **Karl N. Kirschner**: conceptualization; funding acquisition; resources; supervision; visualization; and writing—review & editing.

## Data and Software Availability

The code, all other scripts, and input files used to perform the optimizations conducted herein are available at: https://github.com/rstrickstrock/SMAOpt.git

*Note:* The code is provided for reproduction purposes and may need to be adapted to the IT infrastructure on which it is used.

The MP2/AVTZ optimized geometries for octane are available in Ref. [28] Supporting Information material.

## Supporting information

Supplementary Material

## Data Availability

The data that support the findings of this study are openly available in [a GitHub repository] at [https://github.com/rstrickstrock/SMAOpt.git], reference number [0].
