# Supplementary material for: Speed up Multi‐Scale Force‐Field Parameter Optimization by Substituting Molecular Dynamics Calculations with a Machine Learning Surrogate Model
Source: Chemphyschem. 2025 Sep 5;26(20):e202500353. doi: 10.1002/cphc.202500353 (PMC12530876; doi:10.1002/cphc.202500353)
Supplement: Supplementary file 1 — Supplementary Material [file CPHC-26-e202500353-s001.pdf]

Supporting Information (SI)

Surrogate Model Training

For the reproducibility of the 50 quasi-random splits of the datasets into training and test data the following random seeds are used:
678, 147, 561, 237, 588, 951, 490, 395, 877, 297, 721, 711, 985, 171, 75, 16, 669, 530, 999, 794, 936, 111, 816, 968, 48, 986, 829, 996, 272, 759, 390, 930, 633, 928, 854, 554, 562, 78, 222, 294, 725, 582, 731, 249, 791, 35, 180, 510, 593, 634.

Datasets

Heatmaps

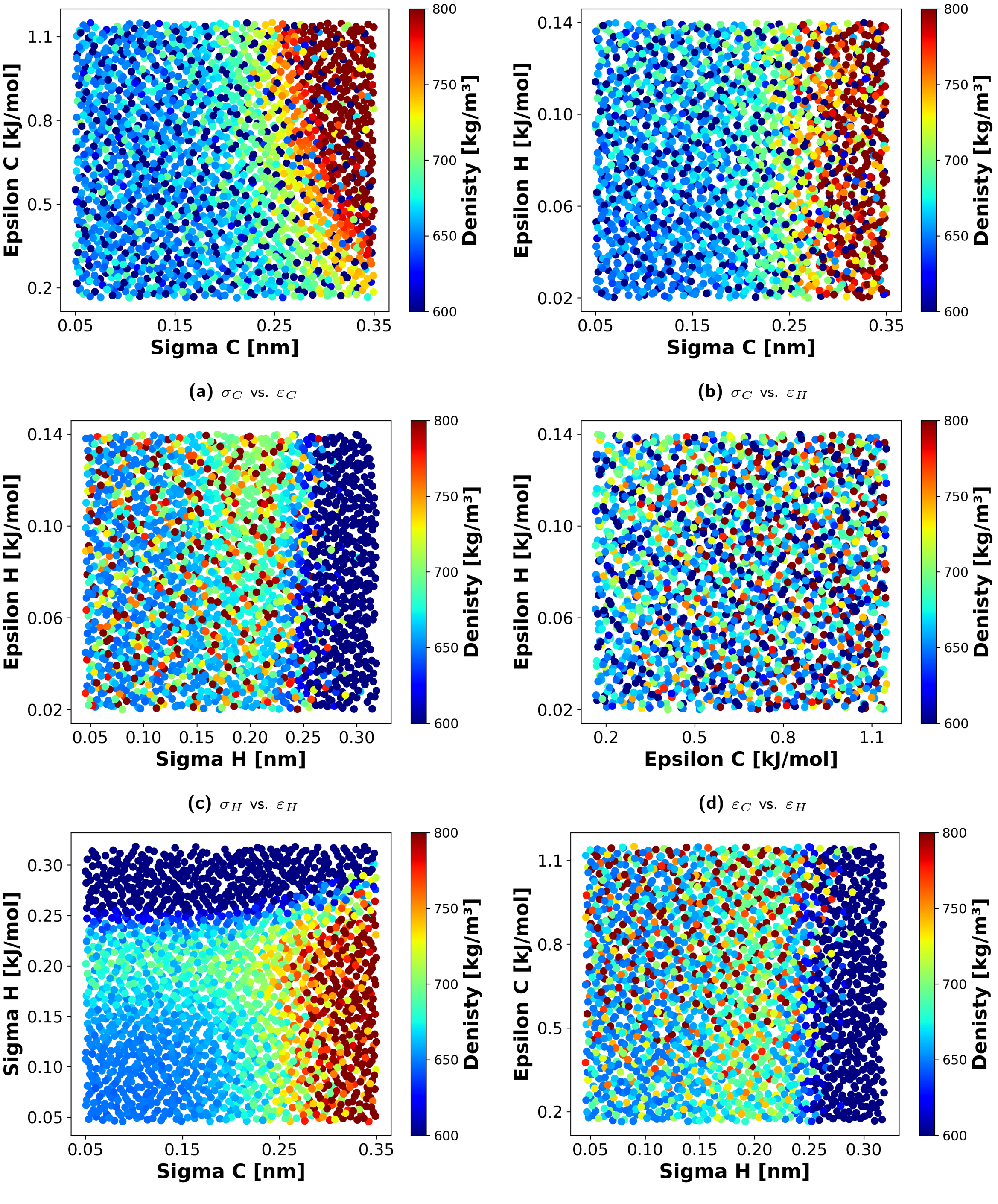

(a) σ_C vs. ε_C **(b)** σ_C vs. ε_H

(c) σ_H vs. ε_H **(d)** ε_C vs. ε_H

(e) σ_C vs. σ_H **(f)** σ_H vs. ε_C

Figure 12. Octane's density for all pairs of the LJPARAMS.

Violin Plots

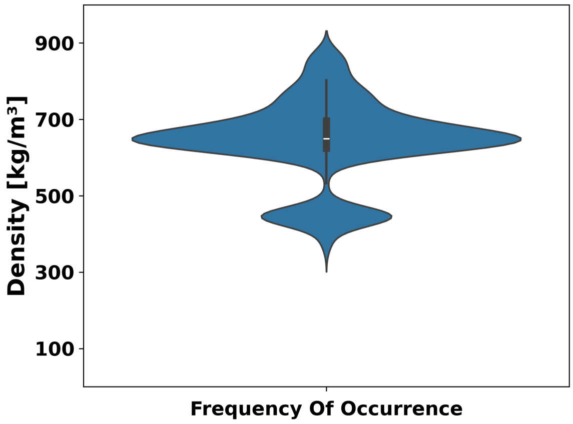

(a) Before preparation.

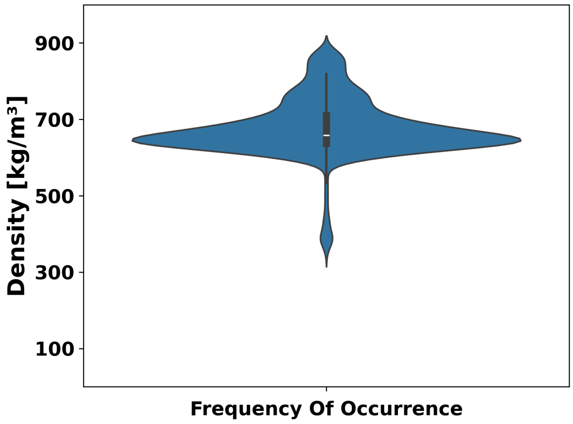

(b) After preparation.

Figure 13. Frequency of occucrance for the density in the "Grid1296" dataset.

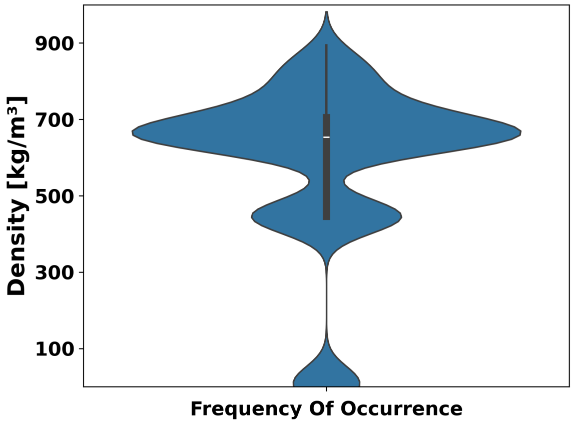

(a) Before preparation.

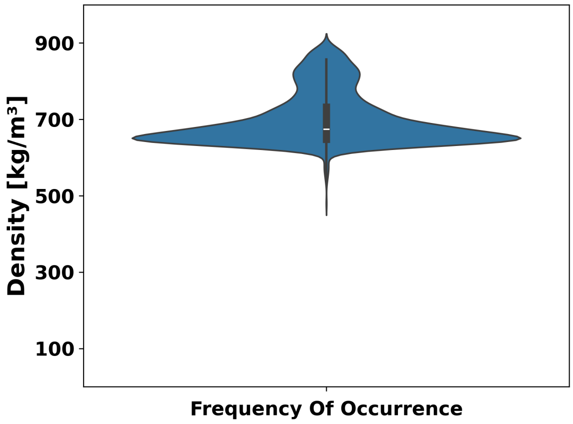

(b) After preparation.

Figure 14. Frequency of occucrance for the density in the "Grid2401" dataset.

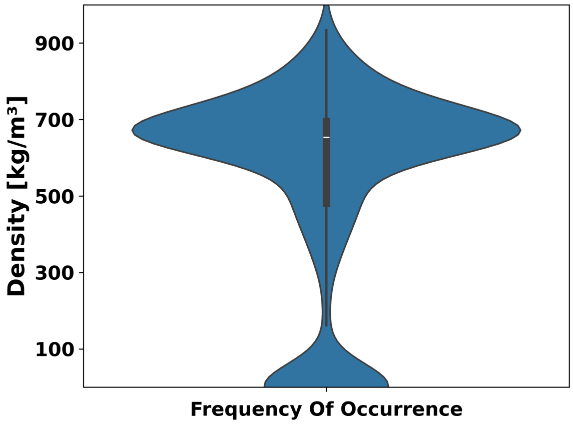

(a) Before preparation.

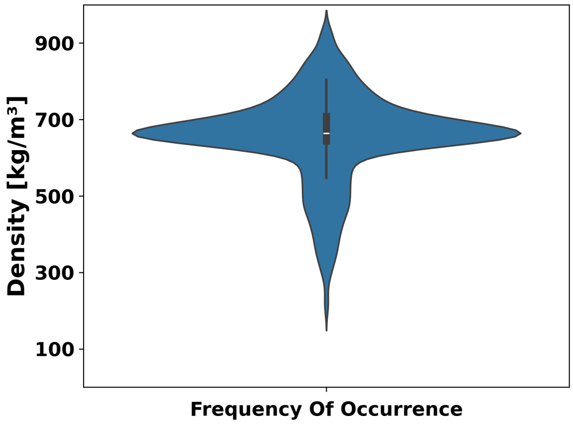

(b) After preparation.

Figure 15. Frequency of occucrance for the density in the "Sobol2" dataset.

Linear Regression

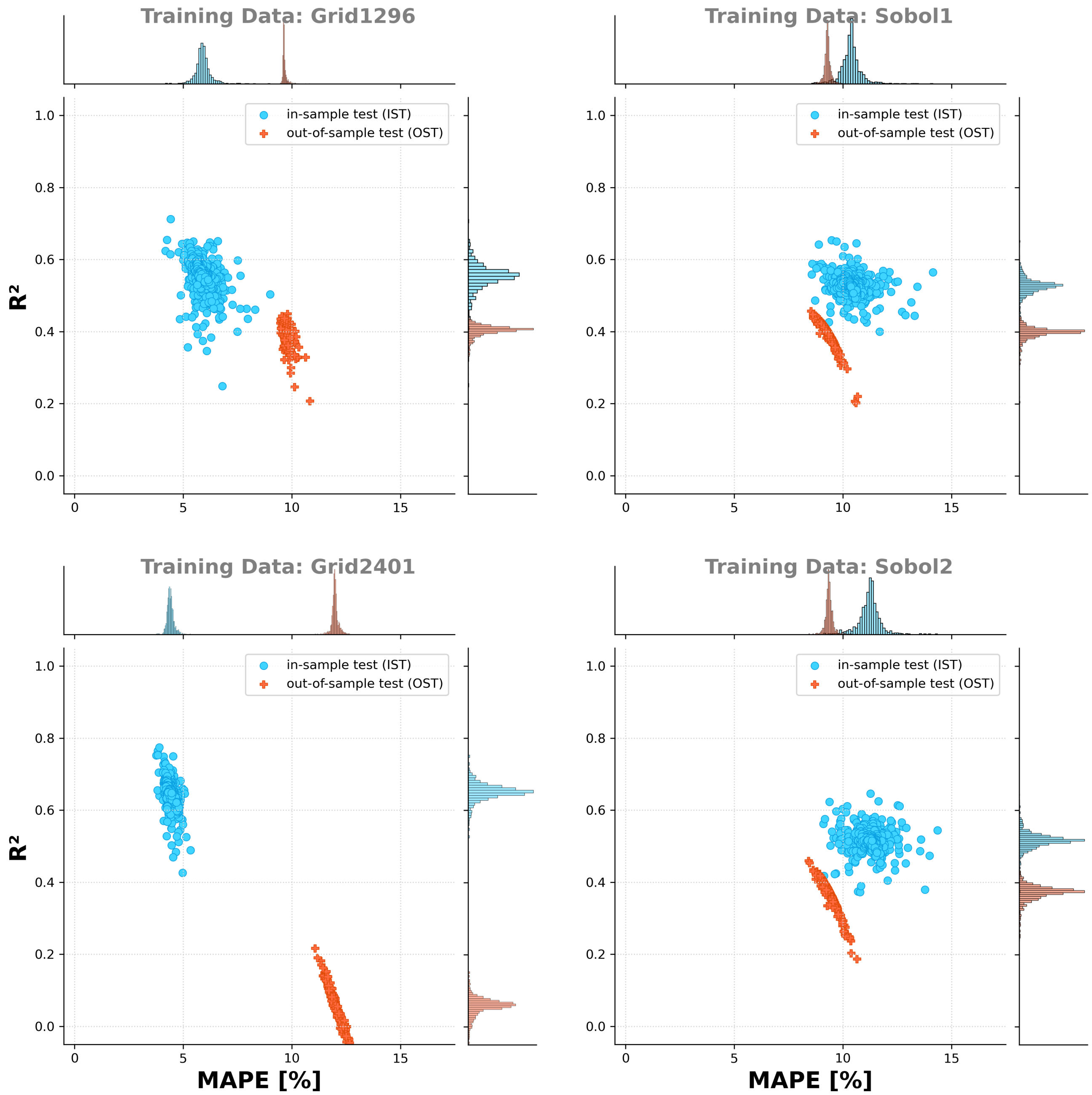

Figure 16. R^2 scores plotted against the MAPE of the linear regression models. Comparing the OST (orange) to the IST (blue) the, models trained using the Grid1296 and Grid2401 (first column) perform noticeably worse than the models trained based on the Sobol1 and Sobol2 datasets (second column). The histograms above and on the right show the distribution of the MAPE and R^2, respectively.

Table 9. Average MAPE and average R^2 values for the predictions given by linear regression models for the four different training datasets. The averages are for all data set ratios and random initial seeds combined.

Dataset	avg. MAPE	avg. R^2
Grid1296 (IST)	0.05915 ± 0.00378	0.55153 ± 0.03808
Grid1296 (OST)	0.09665 ± 0.00110	0.40246 ± 0.01953
Grid2401 (IST)	0.04414 ± 0.00161	0.65026 ± 0.02929
Grid2401 (OST)	0.11998 ± 0.00174	0.05818 ± 0.02910
Sobol1 (IST)	0.10387 ± 0.00497	0.52710 ± 0.02368
Sobol1 (OST)	0.09293 ± 0.00184	0.39690 ± 0.02024
Sobol2 (IST)	0.11220 ± 0.00524	0.51668 ± 0.02477
Sobol2 (OST)	0.09364 ± 0.00201	0.37085 ± 0.02475

Polynomial Regression

Table 10. Avgerage MAPE and average R^2 for the polynomial regression models for the IST. The used polynomial degree is d. The averages are for all data set ratios and random initial seeds combined.

Dataset	d	avg. MAPE	avg. R^2
Grid1296 (IST)	1	0.05920 ± 0.00380	0.55150 ± 0.03810
	2	0.03180 ± 0.00200	0.88900 ± 0.01710
	3	0.01320 ± 0.00550	0.97350 ± 0.03680
	4	0.00960 ± 0.01020	0.96230 ± 0.26290
	5	0.01050 ± 0.01270	0.94540 ± 0.19740
	6	0.01400 ± 0.01550	0.89740 ± 0.31360
	7	0.02120 ± 0.02450	0.69340 ± 1.26740
	8	0.03110 ± 0.03200	0.21910 ± 2.60340
	9	0.05540 ± 0.08610	-8.23030 ± 134.18210
	10	0.07630 ± 0.09490	-13.40070 ± 112.52530
Grid2401 (IST)	1	0.04410 ± 0.00160	0.65030 ± 0.02930
	2	0.02210 ± 0.00180	0.84470 ± 0.03800
	3	0.01960 ± 0.02260	0.76440 ± 1.73360
	4	0.01760 ± 0.02790	0.68910 ± 2.03130
	5	0.02340 ± 0.04820	0.13040 ± 6.10380
	6	0.03510 ± 0.07050	-1.25560 ± 14.29330
	7	0.23410 ± 4.82380	-1.44717e+5 ± 4.39194e+6
	8	0.10370 ± 0.19530	-42.43320 ± 304.38350
	9	0.20250 ± 0.46450	-702.84520 ± 7569.26730
	10	0.25360 ± 0.37110	-491.01380 ± 4569.34680
Sobol1 (IST)	1	0.10390 ± 0.00500	0.52710 ± 0.02370
	2	0.04880 ± 0.00230	0.89730 ± 0.00850
	3	0.01930 ± 0.00690	0.97720 ± 0.03080
	4	0.01750 ± 0.00700	0.97920 ± 0.02930
	5	0.01400 ± 0.01310	0.96970 ± 0.10120
	6	0.01440 ± 0.01950	0.93490 ± 0.23170
	7	0.01690 ± 0.02040	0.90820 ± 0.26800
	8	0.02490 ± 0.03220	0.73590 ± 0.82820
	9	0.04020 ± 0.05410	0.02630 ± 3.95440
	10	0.05580 ± 0.04680	-0.27590 ± 2.49060
Sobol2 (IST)	1	0.11220 ± 0.00520	0.51670 ± 0.02480
	2	0.05130 ± 0.00270	0.89390 ± 0.01120
	3	0.02870 ± 0.01470	0.94470 ± 0.20180
	4	0.02750 ± 0.01690	0.93620 ± 0.20410
	5	0.02080 ± 0.02480	0.90840 ± 0.57220
	6	0.02210 ± 0.02520	0.88810 ± 0.40350
	7	0.02820 ± 0.03220	0.79160 ± 0.60510
	8	0.03760 ± 0.03560	0.64450 ± 0.75440
	9	0.05800 ± 0.05240	0.01080 ± 2.33220
	10	0.08920 ± 0.06430	-1.52390 ± 5.88960

Table 11. Avgerage MAPE and average R^2 for the polynomial regression models for the OST. The used polynomial degree is d. The averages are for all data set ratios and random initial seeds combined.

Dataset	d	avg. MAPE	avg. R^2
Grid1296 (OST)	1	0.09670 ± 0.00110	0.40250 ± 0.01950
	2	0.05830 ± 0.00210	0.77940 ± 0.02160
	3	0.03030 ± 0.00600	0.91850 ± 0.03940
	4	0.04690 ± 0.01060	0.71620 ± 0.29200
	5	0.06030 ± 0.01470	0.43920 ± 0.40540
	6	2.11541e+6 ± 4.19603e+6	-3.96959e+15 ± 1.95012e+16
	7	1.50042e+6 ± 3.89729e+6	-3.42860e+15 ± 2.14335e+16
	8	2.29604e+6 ± 8.77021e+6	-1.93422e+16 ± 2.96841e+17
	9	7.46428e+6 ± 4.47347e+6	-5.72522e+17 ± 5.08630e+18
	10	4.34143e+6 ± 3.683871e+6	-4.26776e+17 ± 6.08927e+18
Grid2401 (OST)	1	0.12000 ± 0.00170	0.05820 ± 0.02910
	2	0.09180 ± 0.00340	0.36970 ± 0.05160
	3	0.05620 ± 0.02790	0.74040 ± 0.98590
	4	0.04680 ± 0.03810	0.61340 ± 1.39660
	5	0.10230 ± 0.08980	-1.31970 ± 7.44620
	6	0.21860 ± 0.27640	-19.26720 ± 77.79550
	7	2.57947e+5 ± 1.37519e+6	-1.43517e+14 ± 1.81620e+15
	8	1.89752e+5 ± 1.07027e+6	-9.47944e+13 ± 1.83858e+15
	9	1.01381e+5 ± 2.35872e+5	-6.30234e+12 ± 5.73940e+13
	10	4.32293e+5 ± 2.17519e+6	-5.31050e+14 ± 9.84293e+15
Sobol1 (OST)	1	0.09290 ± 0.00180	0.39690 ± 0.02020
	2	0.05010 ± 0.00100	0.80930 ± 0.00980
	3	0.02680 ± 0.00710	0.89640 ± 0.06360
	4	0.02460 ± 0.00770	0.90470 ± 0.07130
	5	0.02190 ± 0.01510	0.88330 ± 0.27970
	6	0.02520 ± 0.02420	0.74300 ± 0.81810
	7	0.02950 ± 0.02580	0.60910 ± 1.12530
	8	0.04280 ± 0.04860	-0.36920 ± 4.68730
	9	0.06940 ± 0.08870	-4.69980 ± 22.27100
	10	0.09880 ± 0.08010	-7.57610 ± 17.11390
Sobol2 (OST)	1	0.09360 ± 0.00200	0.37090 ± 0.02480
	2	0.04910 ± 0.00110	0.81320 ± 0.01150
	3	0.02670 ± 0.01360	0.89190 ± 0.30660
	4	0.02640 ± 0.01470	0.87680 ± 0.31520
	5	0.02240 ± 0.02270	0.81150 ± 1.21860
	6	0.02350 ± 0.02190	0.76690 ± 0.85080
	7	0.02950 ± 0.02790	0.54930 ± 1.28140
	8	0.03740 ± 0.03180	0.14040 ± 1.91330
	9	0.05500 ± 0.04520	-1.39790 ± 4.98690
	10	0.08260 ± 0.05370	-5.31710 ± 11.15940

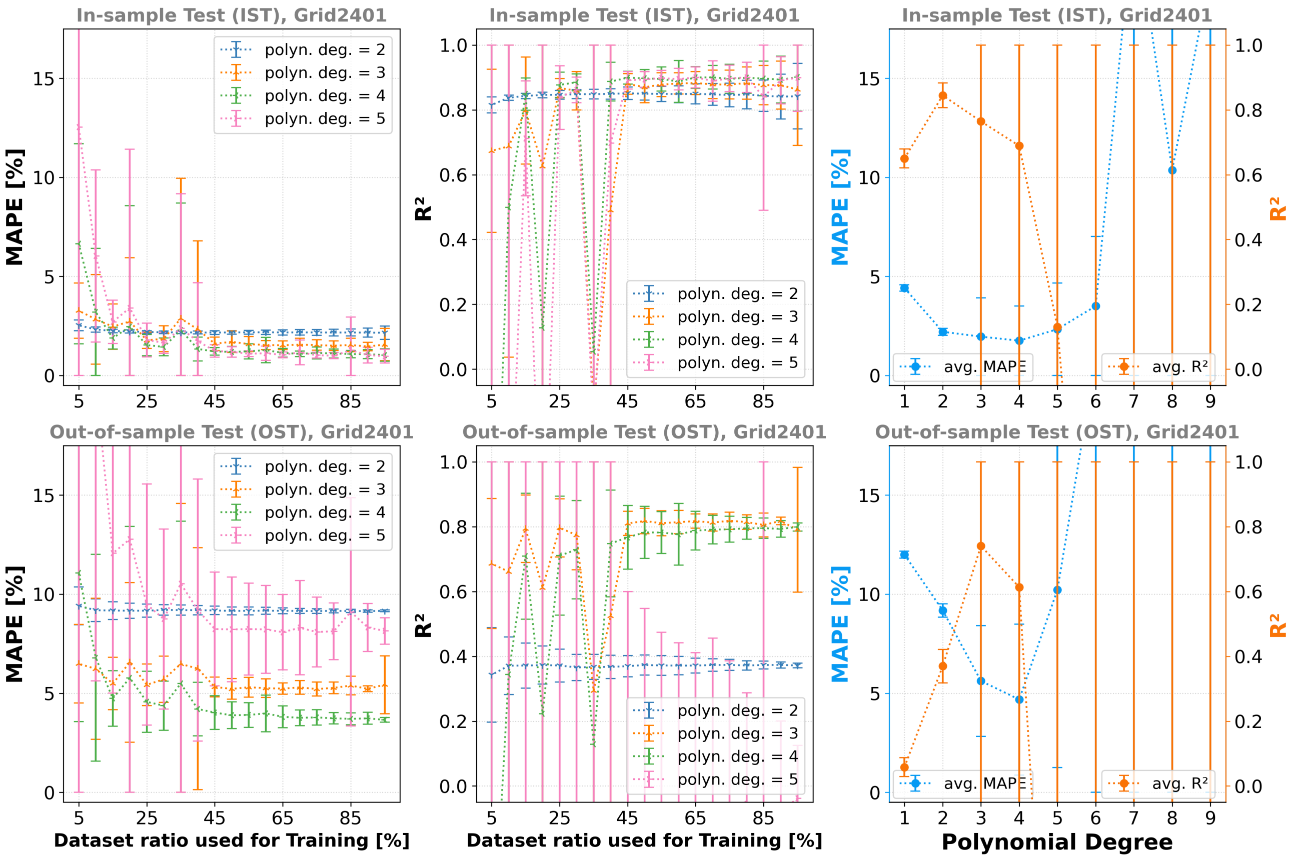

Figure 17. First and second column: avg. MAPE and avg. R^2 scores for the different ratios of the datasets. Plotted are the averages for the most relevant polynomial degrees d. For each ratio 50 different training data splits (using different random seeds) are averaged. Third column: MAPE and R^2 score averaged over all models trained for the corresponding polynomial degree d. First row: IST. Second row: OST. Dataset: Grid2401.

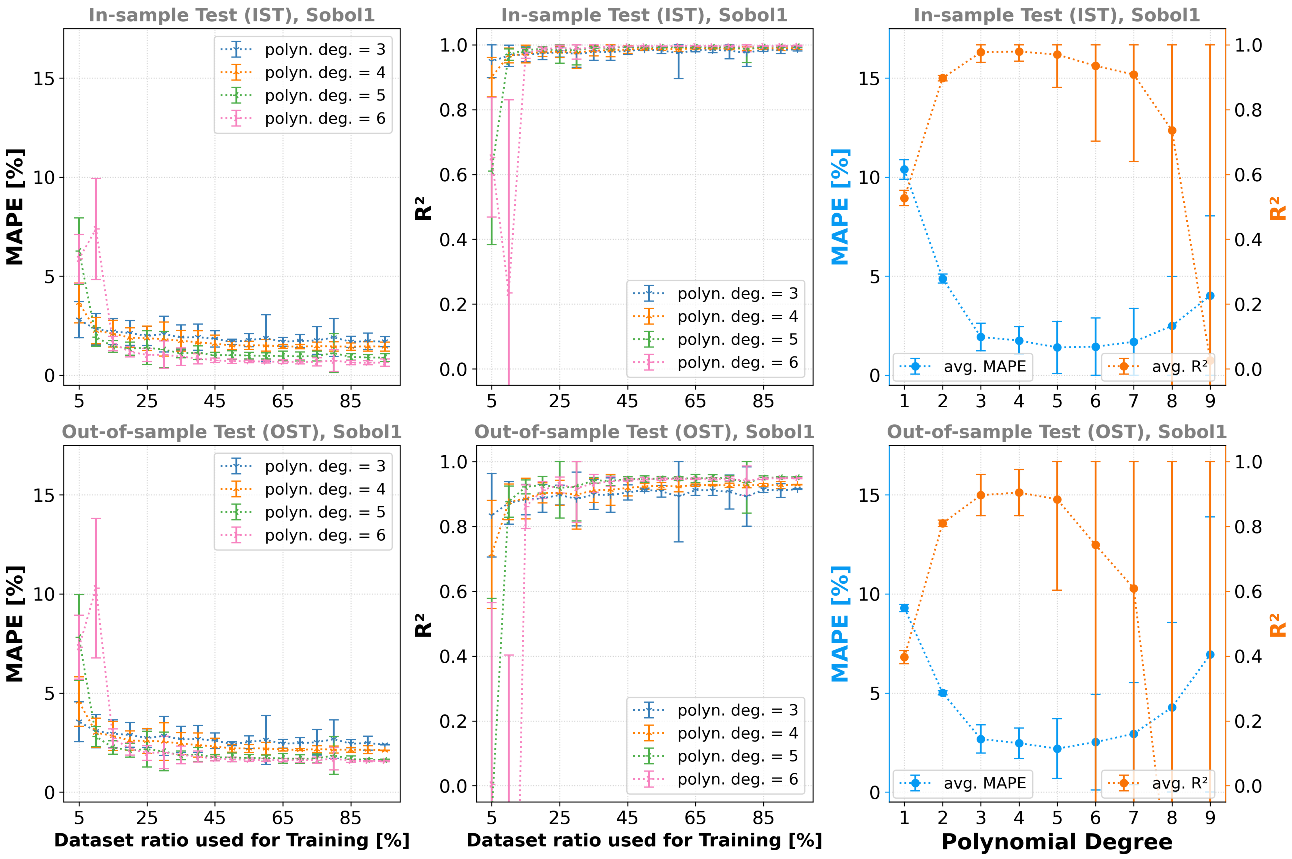

Figure 18. First and second column: avg. MAPE and avg. R^2 scores for the different ratios of the datasets. Plotted are the averages for the most relevant polynomial degrees d. For each ratio 50 different training data splits (using different random seeds) are averaged. Third column: MAPE and R^2 score averaged over all models trained for the corresponding polynomial degree d. First row: IST. Second row: OST. Dataset: Sobol1.

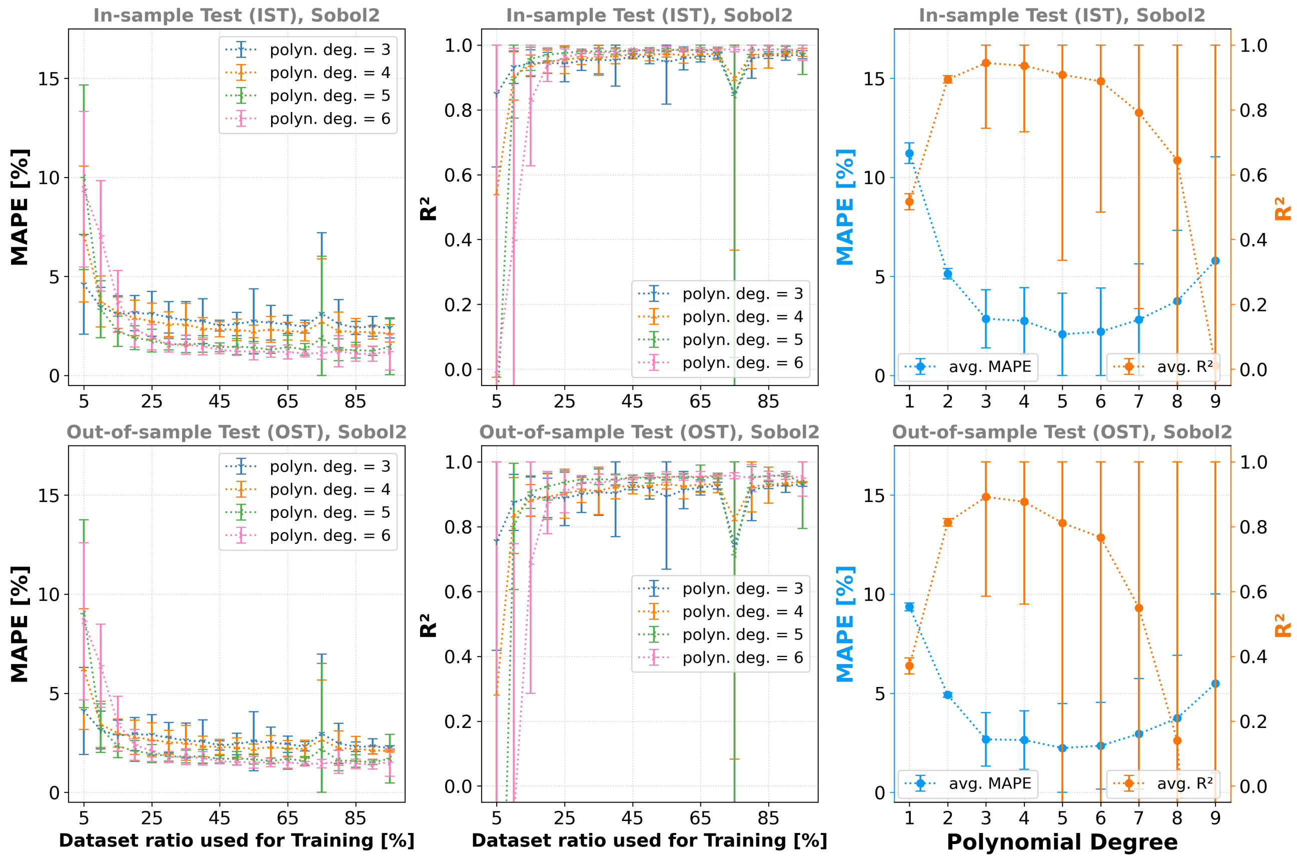

Figure 19. First and second column: avg. MAPE and avg. R^2 scores for the different ratios of the datasets. Plotted are the averages for the most relevant polynomial degrees d. For each ratio 50 different training data splits (using different random seeds) are averaged. Third column: MAPE and R^2 score averaged over all models trained for the corresponding polynomial degree d. First row: IST. Second row: OST. Dataset: Sobol2.

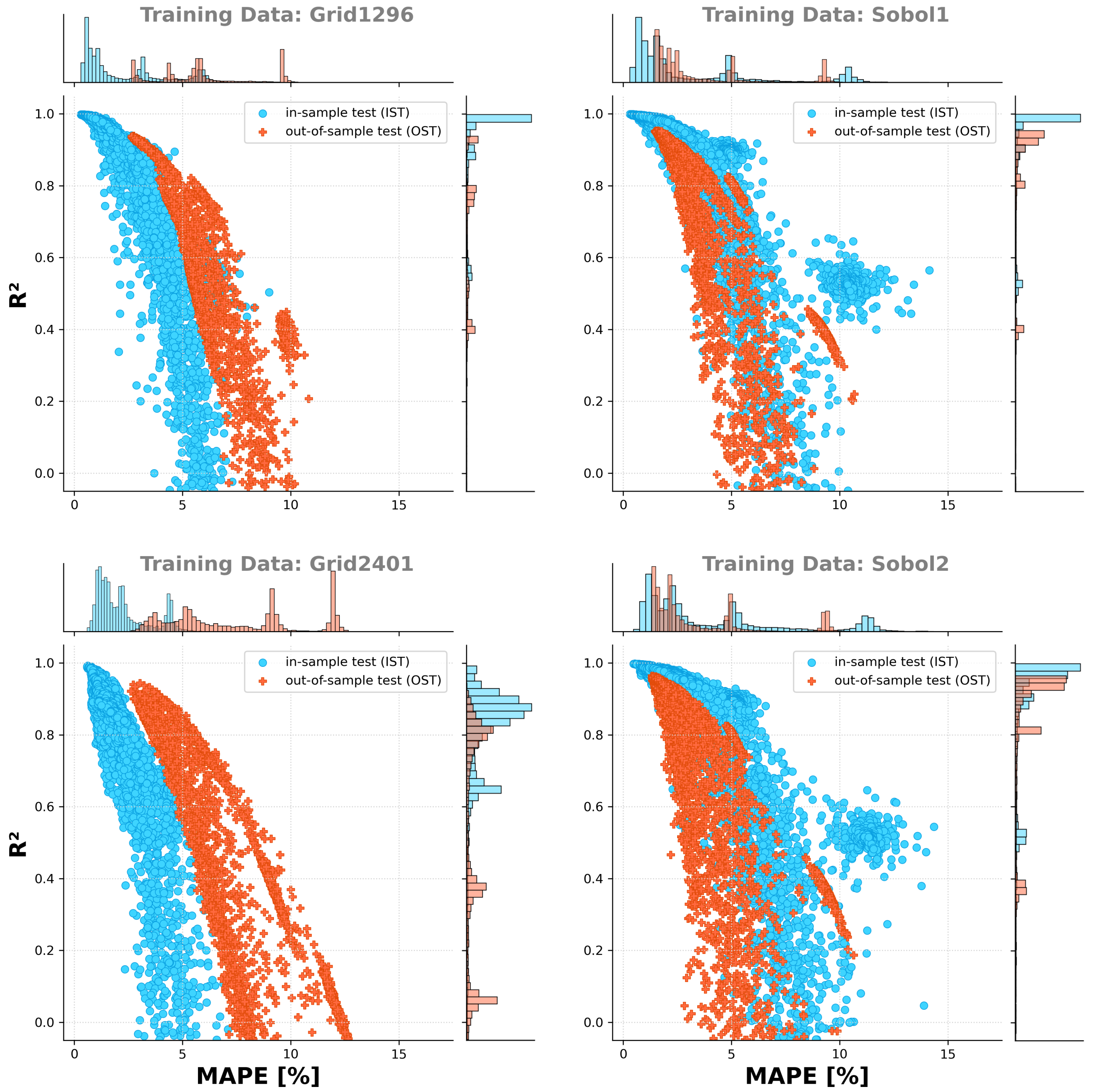

Figure 20. R^2 scores plotted against the MAPE of the polynomial regression models. Comparing the OST (orange) to the IST (blue) the, models trained using the Grid1296 and Grid2401 (first column) perform noticeably worse than the models trained based on the Sobol1 and Sobol2 datasets (second column). The histograms above and on the right show the distribution of the MAPE and R^2, respectively.

Random Forest Regression

Table 12. Avgerage MAPE and average R^2 for the random forest regression models for the IST. The number of trees is #t. The averages are for all data set ratios and random initial seeds combined.

Dataset	#t	avg. MAPE	avg. R^2
Grid1296 (IST)	10	0.00960 ± 0.00530	0.97290 ± 0.04180
	100	0.00900 ± 0.00490	0.97580 ± 0.03890
	250	0.00900 ± 0.00490	0.97600 ± 0.03860
	500	0.00900 ± 0.00490	0.97610 ± 0.03850
	750	0.00900 ± 0.00490	0.97610 ± 0.03860
	1000	0.00900 ± 0.00490	0.97610 ± 0.03850
Grid2401 (IST)	10	0.01250 ± 0.00320	0.84650 ± 0.05110
	100	0.01190 ± 0.00290	0.85710 ± 0.04420
	250	0.01190 ± 0.00290	0.85760 ± 0.04400
	500	0.01190 ± 0.00290	0.85780 ± 0.04420
	750	0.01190 ± 0.00290	0.85780 ± 0.04420
	1000	0.01190 ± 0.00290	0.85780 ± 0.04420
Sobol1 (IST)	10	0.01830 ± 0.00600	0.96990 ± 0.02110
	100	0.01670 ± 0.00570	0.97410 ± 0.01890
	250	0.01660 ± 0.00570	0.97440 ± 0.01870
	500	0.01650 ± 0.00570	0.97450 ± 0.01860
	750	0.01650 ± 0.00570	0.97460 ± 0.01860
	1000	0.01650 ± 0.00570	0.97460 ± 0.01860
Sobol2 (IST)	10	0.02360 ± 0.00690	0.95530 ± 0.02510
	100	0.02180 ± 0.00660	0.96090 ± 0.02280
	250	0.02160 ± 0.00660	0.96130 ± 0.02260
	500	0.02160 ± 0.00660	0.96140 ± 0.02260
	750	0.02160 ± 0.00660	0.96150 ± 0.02250
	1000	0.02160 ± 0.00660	0.96150 ± 0.02250

Table 13. Avgerage MAPE and average R^2 for the random forest regression models for the OST. The number of trees is #t. The averages are for all data set ratios and random initial seeds combined.

Dataset	# Trees	avg. MAPE	avg. R^2
Grid1296 (OST)	10	0.04680 ± 0.00480	0.82220 ± 0.05310
	100	0.04650 ± 0.00450	0.82490 ± 0.05120
	250	0.04650 ± 0.00450	0.82510 ± 0.05090
	500	0.04650 ± 0.00450	0.82520 ± 0.05080
	750	0.04650 ± 0.00450	0.82520 ± 0.05080
	1000	0.04650 ± 0.00450	0.82520 ± 0.05080
Grid2401 (OST)	10	0.09400 ± 0.00360	0.31830 ± 0.04840
	100	0.09380 ± 0.00350	0.32150 ± 0.04710
	250	0.09380 ± 0.00350	0.32160 ± 0.04700
	500	0.09380 ± 0.00350	0.32160 ± 0.04700
	750	0.09380 ± 0.00350	0.32160 ± 0.04700
	1000	0.09380 ± 0.00350	0.32160 ± 0.04700
Sobol1 (OST)	10	0.02110 ± 0.00390	0.93190 ± 0.02070
	100	0.02020 ± 0.00360	0.93630 ± 0.01810
	250	0.02010 ± 0.00360	0.93660 ± 0.01780
	500	0.02010 ± 0.00360	0.93670 ± 0.01780
	750	0.02010 ± 0.00360	0.93680 ± 0.01780
	1000	0.02010 ± 0.00360	0.93680 ± 0.01770
Sobol2 (OST)	10	0.01990 ± 0.00470	0.93970 ± 0.02590
	100	0.01870 ± 0.00440	0.94550 ± 0.02320
	250	0.01860 ± 0.00440	0.94590 ± 0.02290
	500	0.01860 ± 0.00440	0.94600 ± 0.02290
	750	0.01860 ± 0.00440	0.94610 ± 0.02280
	1000	0.01860 ± 0.00440	0.94610 ± 0.02280

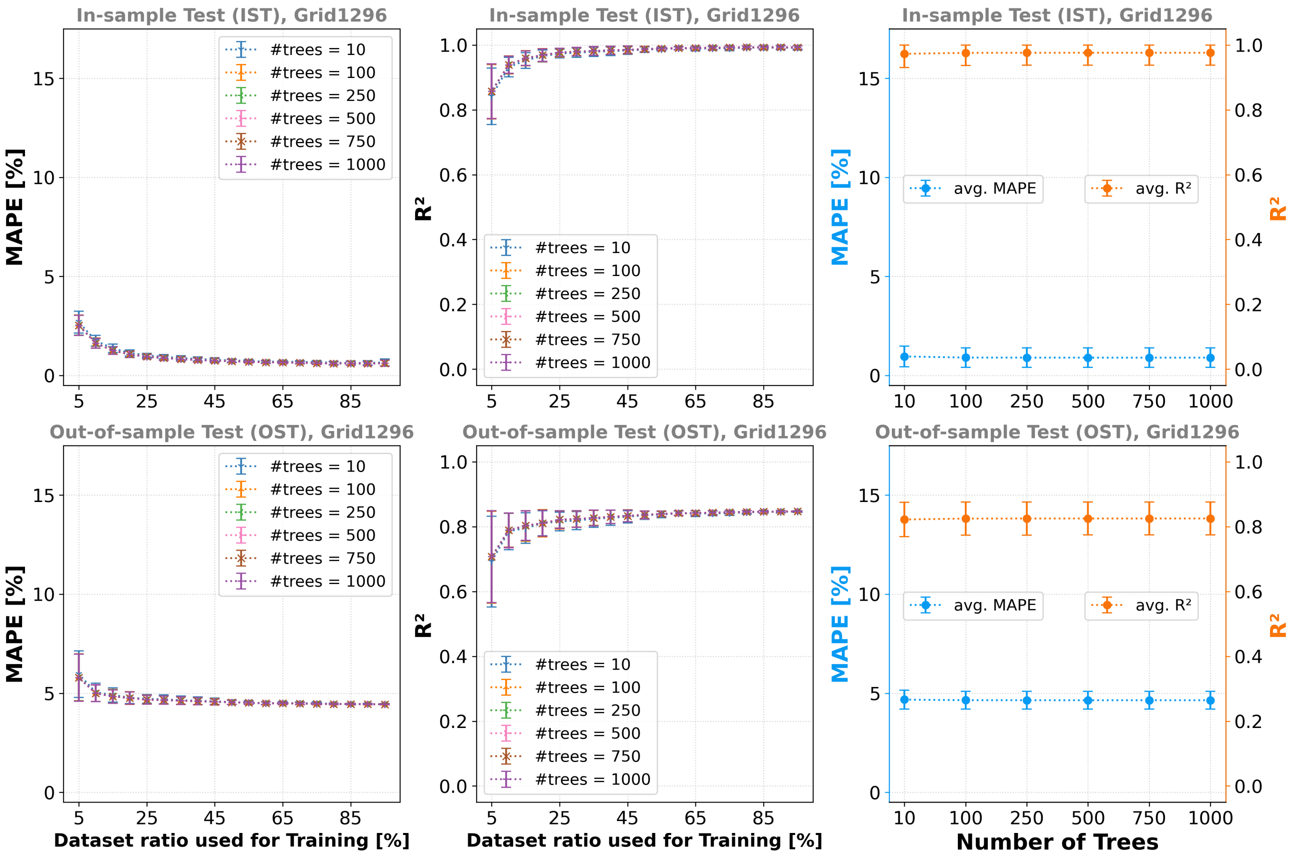

Figure 21. First and second column: avg. MAPE and avg. R^2 scores for different the ratios of the dataset and the different numbers of trees used for the model training. For each ratio 50 different training data splits (using different random seeds) are averaged. Third column: MAPE and R^2 score averaged over all models trained for the corresponding number of trees. Here, no under- or overfitting can be seen. First row: IST. Second row: OST. Dataset: Grid1296.

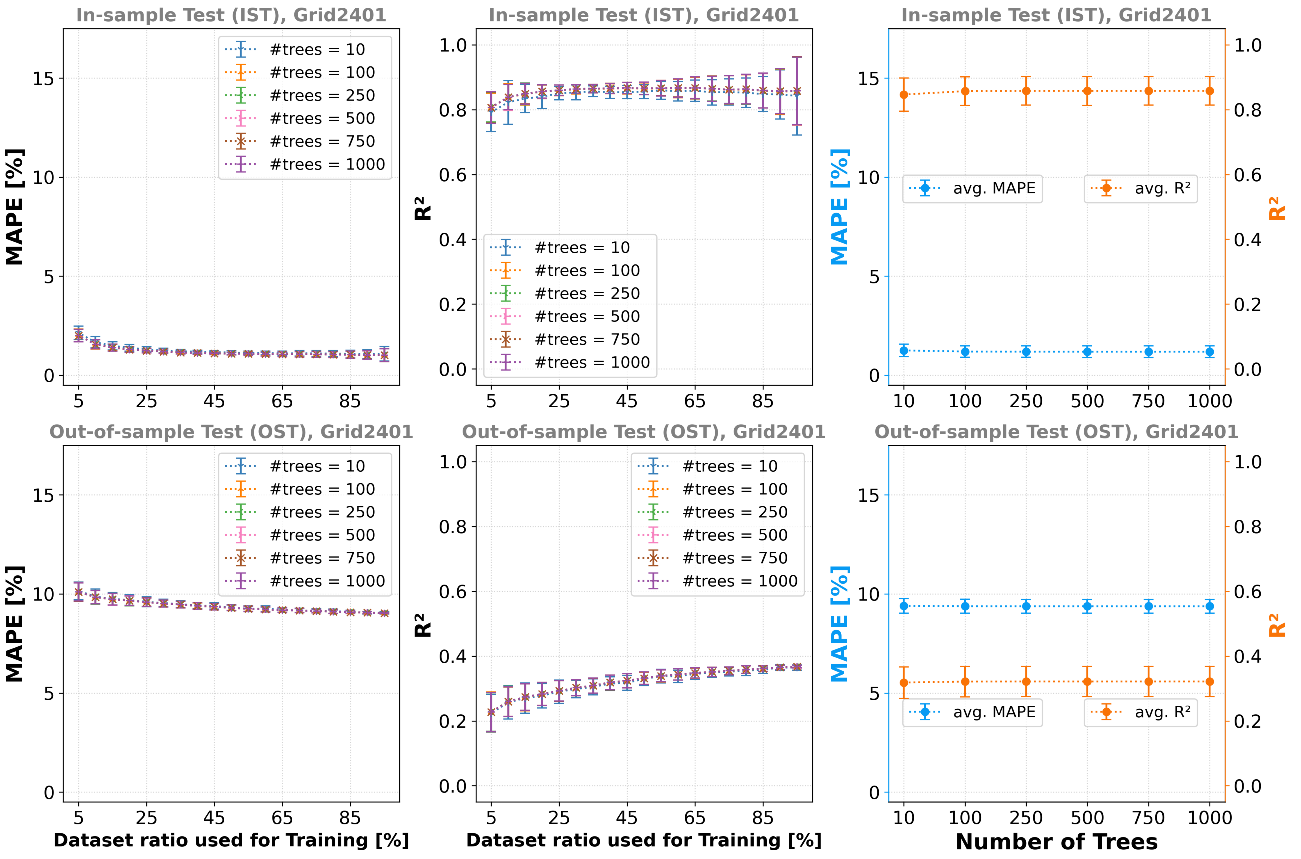

Figure 22. First and second column: avg. MAPE and avg. R^2 scores for different the ratios of the dataset and the different numbers of trees used for the model training. For each ratio 50 different training data splits (using different random seeds) are averaged. Third column: MAPE and R^2 score averaged over all models trained for the corresponding number of trees. Here, no under- or overfitting can be seen. First row: IST. Second row: OST. Dataset: Grid2401.

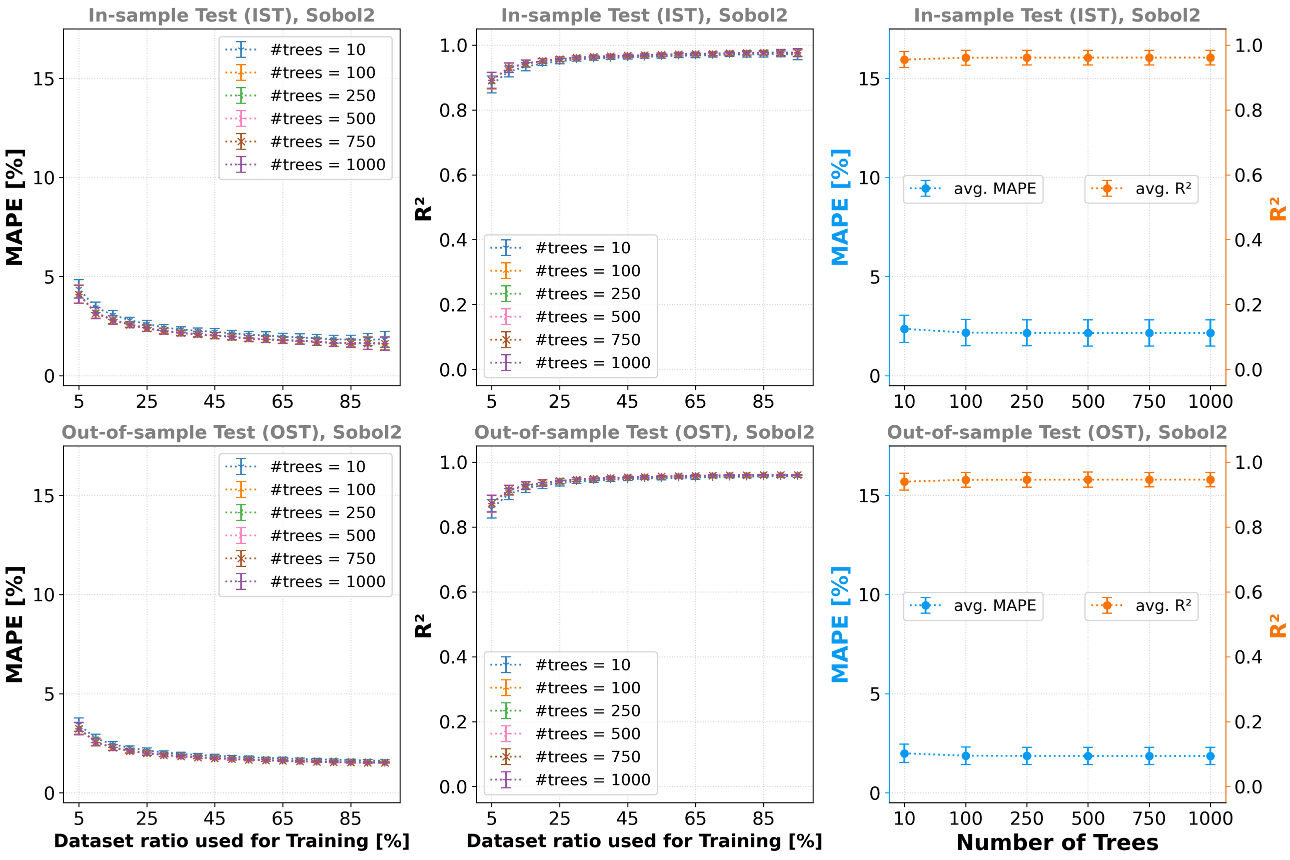

Figure 23. First and second column: avg. MAPE and avg. R^2 scores for different the ratios of the dataset and the different numbers of trees used for the model training. For each ratio 50 different training data splits (using different random seeds) are averaged. Third column: MAPE and R^2 score averaged over all models trained for the corresponding number of trees. Here, no under- or overfitting can be seen. First row: IST. Second row: OST. Dataset: Sobol2.

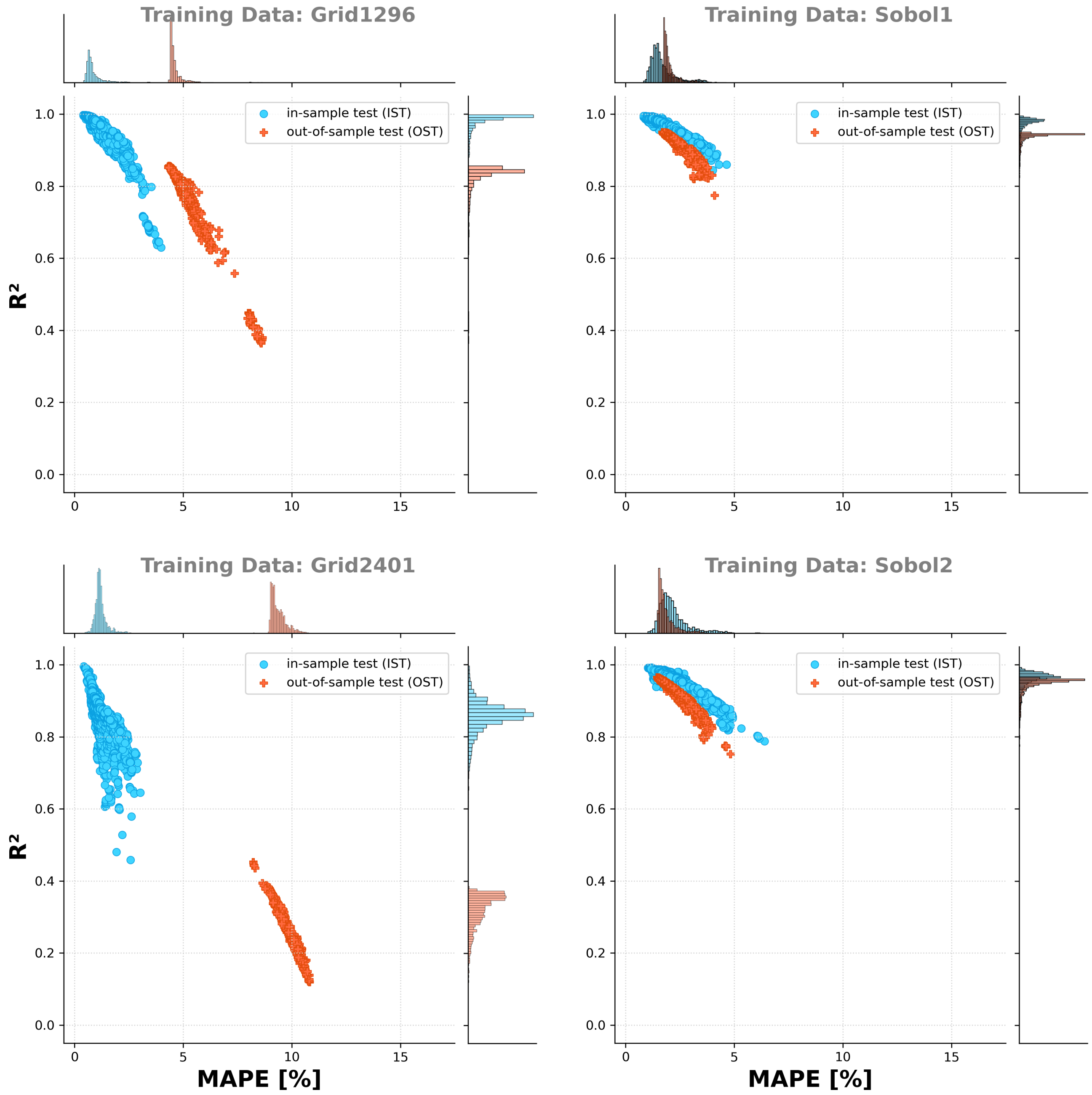

Figure 24. R^2 scores plotted against the MAPE of the random forest regression models. Comparing the OST (orange) to the IST (blue) the, models trained using the Grid1296 and Grid2401 (first column) perform noticeably worse than the models trained based on the Sobol1 and Sobol2 datasets (second column). The histograms above and on the right show the distribution of the MAPE and R^2, respectively.

Gaussian Process Regression

Table 14. Avgerage MAPE and average R^2 for the Gaussian process regression models for the IST. The averages are for all data set ratios and random initial seeds combined.

Dataset	Kernel	avg. MAPE	avg. R^2
Grid1296 (IST)	RBF	0.25180 ± 0.41210	-17.51110 ± 33.60290
	Matérn	0.04240 ± 0.16400	-1.46380 ± 14.16970
	RQ	0.02300 ± 0.01070	0.86340 ± 0.11660
Grid2401 (IST)	RBF	0.32610 ± 0.41880	-29.31660 ± 46.37580
	Matérn	0.07180 ± 0.21890	-4.78690 ± 23.49370
	RQ	0.01840 ± 0.00480	0.84180 ± 0.05570
Sobol1 (IST)	RBF	0.21480 ± 0.39020	-6.35770 ± 14.76400
	Matérn	0.04930 ± 0.17500	-0.29500 ± 6.89610
	RQ	0.02750 ± 0.01710	0.93190 ± 0.08070
Sobol2 (IST)	RBF	0.20510 ± 0.37270	-5.17180 ± 13.04930
	Matérn	0.05480 ± 0.17120	-0.10010 ± 5.81890
	RQ	0.03560 ± 0.01920	0.90670 ± 0.09360

Table 15. Avgerage MAPE and average R^2 for the gaussian process regression models for the OST. The averages are for all data set ratios and random initial seeds combined.

Dataset	Kernel	avg. MAPE	avg. R^2
Grid1296 (OST)	RBF	0.45670 ± 0.33270	-14.49660 ± 16.57690
	Matérn	0.08920 ± 0.15590	-0.62640 ± 7.28420
	RQ	0.06830 ± 0.00980	0.66590 ± 0.11030
Grid2401 (OST)	RBF	0.59320 ± 0.32480	-18.68500 ± 14.81600
	Matérn	0.10120 ± 0.21230	-1.30620 ± 8.97950
	RQ	0.09840 ± 0.00380	0.25540 ± 0.03800
Sobol1 (OST)	RBF	0.22050 ± 0.38730	-9.99300 ± 21.84760
	Matérn	0.05430 ± 0.17400	-0.87640 ± 9.75330
	RQ	0.02690 ± 0.01250	0.89480 ± 0.08910
Sobol2 (OST)	RBF	0.20420 ± 0.37300	-9.29860 ± 21.62580
	Matérn	0.05380 ± 0.17120	-0.87140 ± 9.85720
	RQ	0.02700 ± 0.01340	0.89080 ± 0.10420

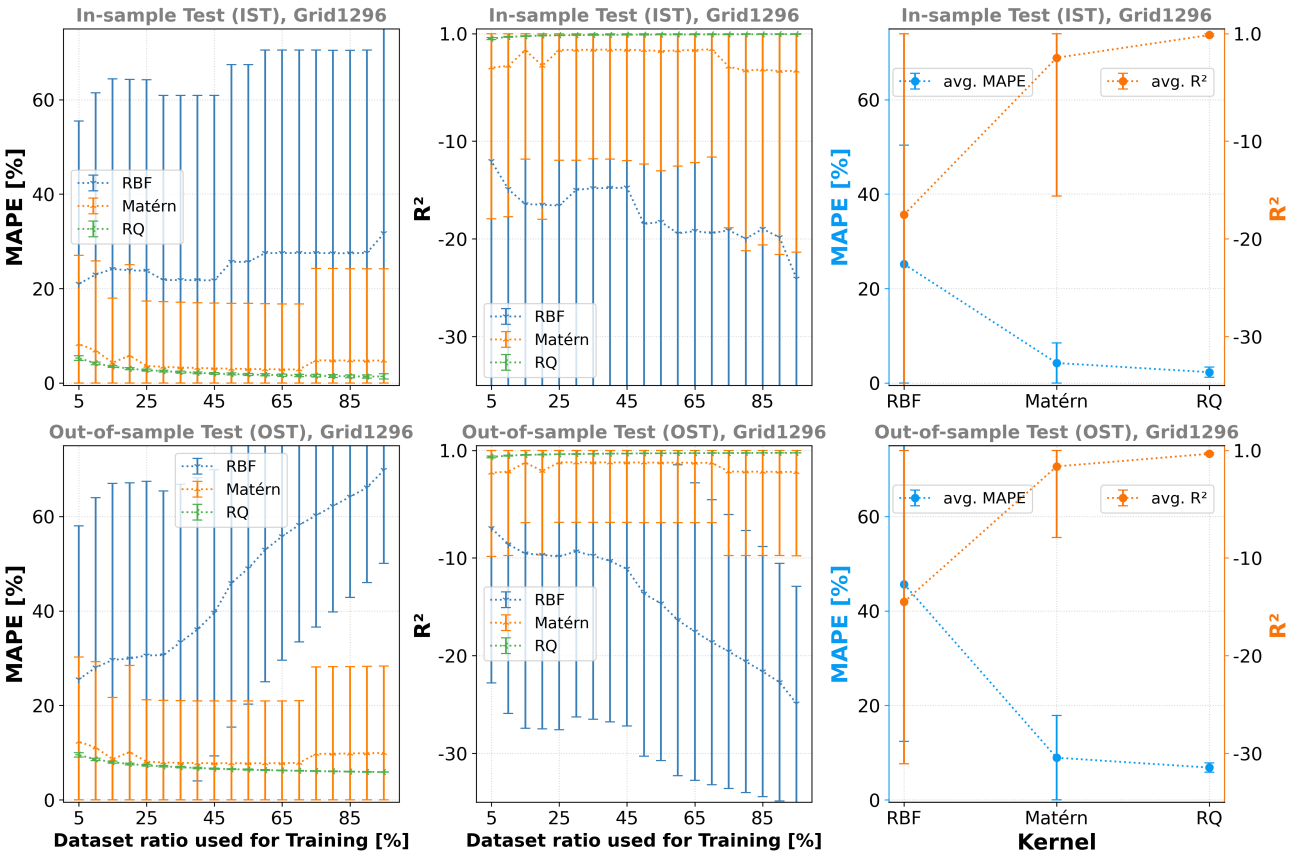

Figure 25. First and second column: avg. MAPE and avg. R^2 scores for different the ratios of the dataset and the different kernels used for the model training. For each ratio 50 different training data splits (using different random seeds) are averaged. The average performance of the RBF and Matérn is worse than for the RQ kernel. However, their variance is very large and well performing models exist. Third column: MAPE and R^2 score averaged over all models trained for the corresponding kernel. First row: IST. Second row: OST. Dataset: Grid1296.

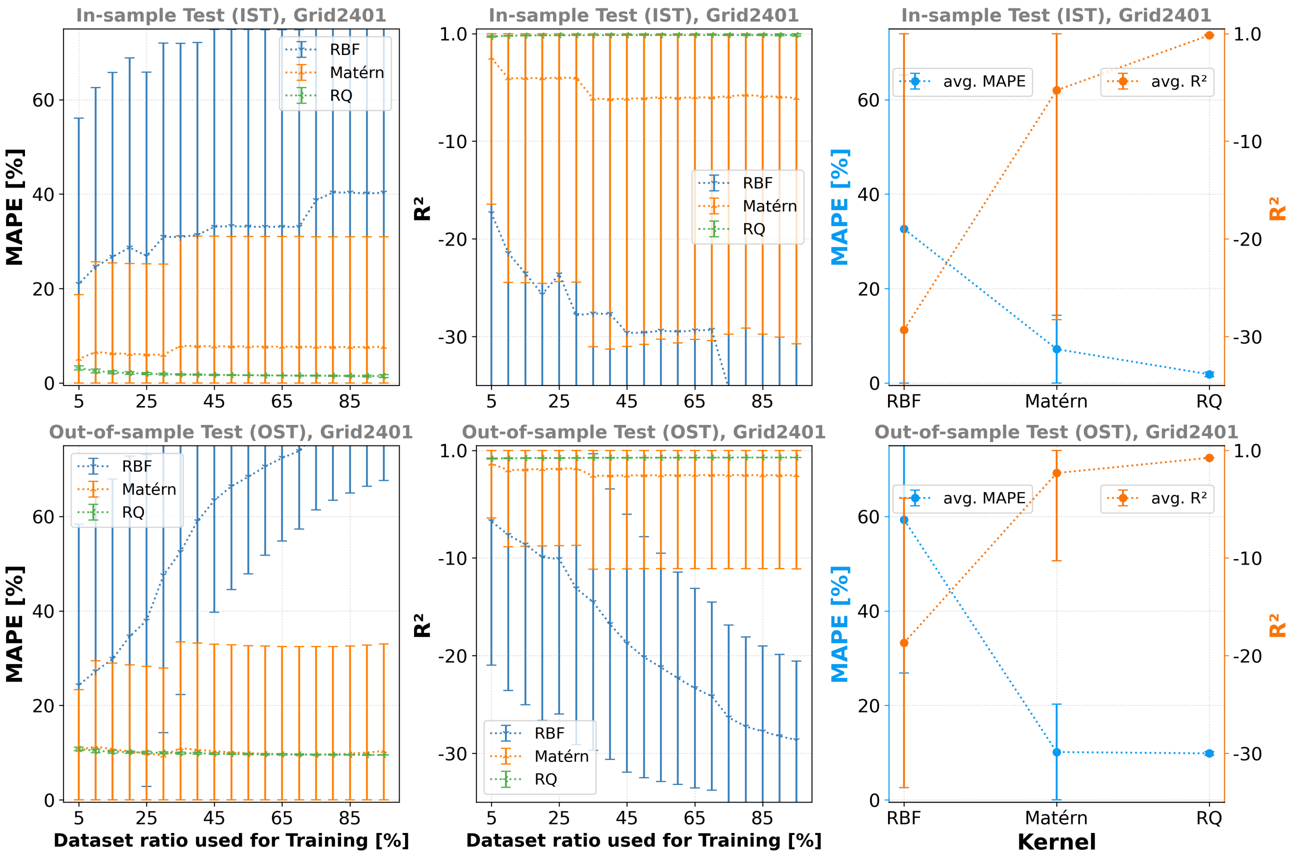

Figure 26. First and second column: avg. MAPE and avg. R^2 scores for different the ratios of the dataset and the different kernels used for the model training. For each ratio 50 different training data splits (using different random seeds) are averaged. The average performance of the RBF and Matérn is worse than for the RQ kernel. However, their variance is very large and well performing models exist. Third column: MAPE and R^2 score averaged over all models trained for the corresponding kernel. First row: IST. Second row: OST. Dataset: Grid2401.

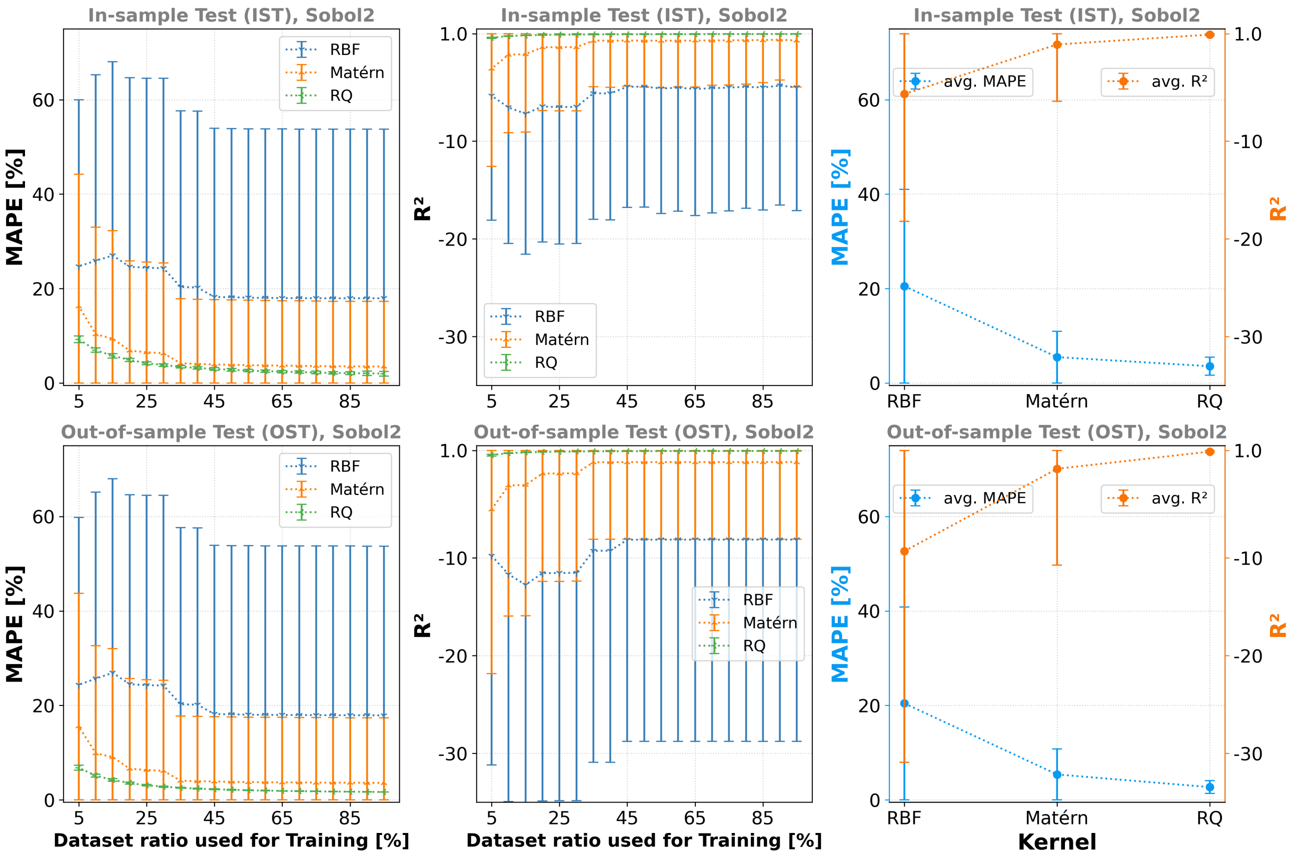

Figure 27. First and second column: avg. MAPE and avg. R^2 scores for different the ratios of the dataset and the different kernels used for the model training. For each ratio 50 different training data splits (using different random seeds) are averaged. The average performance of the RBF and Matérn is worse than for the RQ kernel. However, their variance is very large and well performing models exist. Third column: MAPE and R^2 score averaged over all models trained for the corresponding kernel. First row: IST. Second row: OST. Dataset: Sobol2.

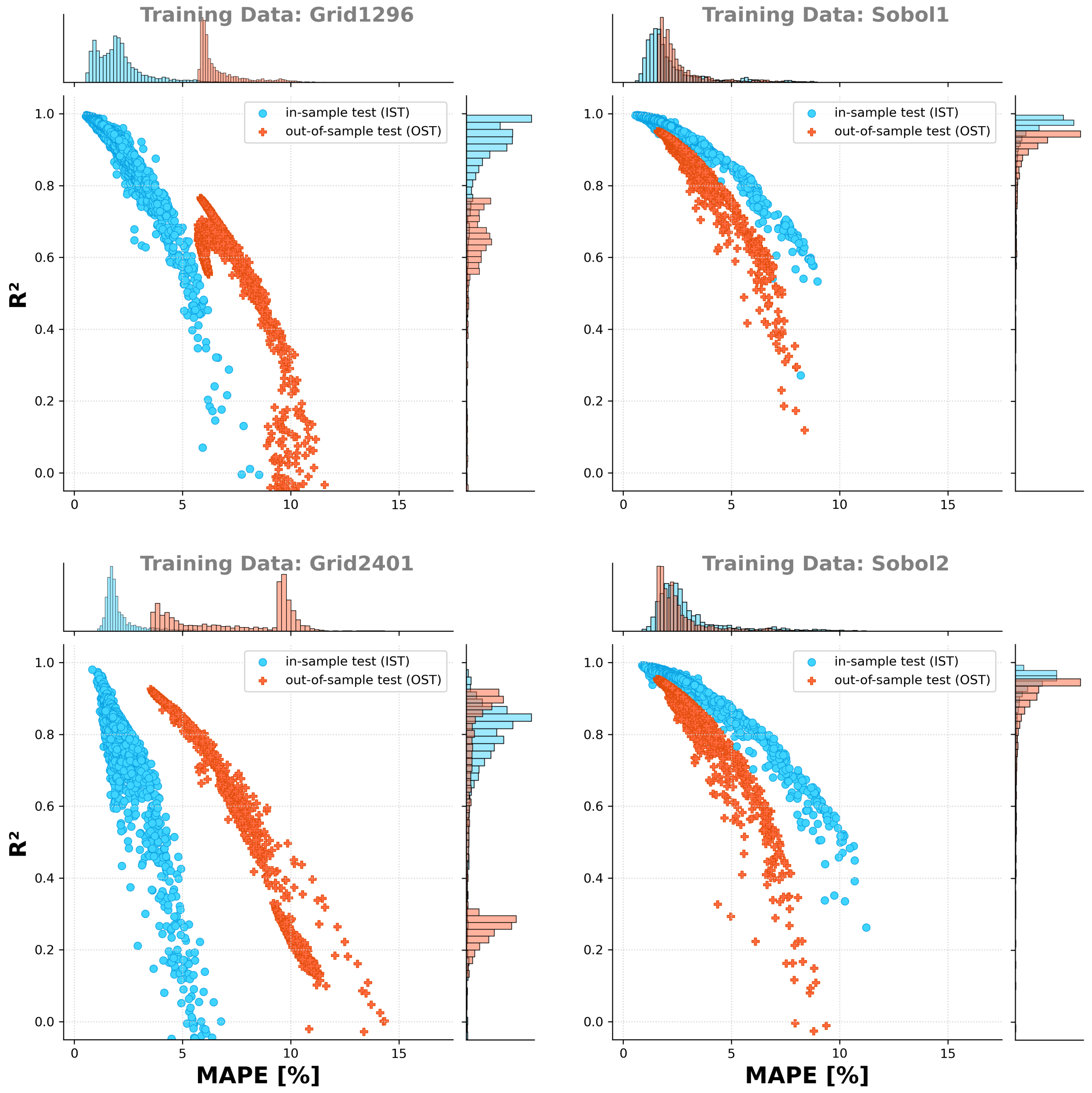

Figure 28. R^2 scores plotted against the MAPE of the Gaussian Process regression models. Comparing the OST (orange) to the IST (blue) the, models trained using the Grid1296 and Grid2401 (first column) perform noticeably worse than the models trained based on the Sobol1 and Sobol2 datasets (second column). The histograms above and on the right show the distribution of the MAPE and R^2, respectively.

Neural Network Regression

23

graphics/Trainings/NeuralNetworks/Ratios-vs-MAPE_R2_NN.png

Figure 29. Avg. MAPE and avg. R^2 scores for different ratios of the datasets used for the model training. For each ratio 50 different splits (based on different random seeds) are averaged. First row: IST. Second row: OST.

Table 16. Used FNN architecture and hyperparameters.

number of nodes	$n_{in} = 4$<br>$n_{h1} = 128$<br>$n_{h2} = 64$<br>$n_{h3} = 32$<br>$n_{out} = 1$
learning cycles	$n_{epochs} = 200$
learning rate	$lr = 0.001$
activation function	LeakyReLU()
loss function	L1Loss()
optimizer	optim.Adam()

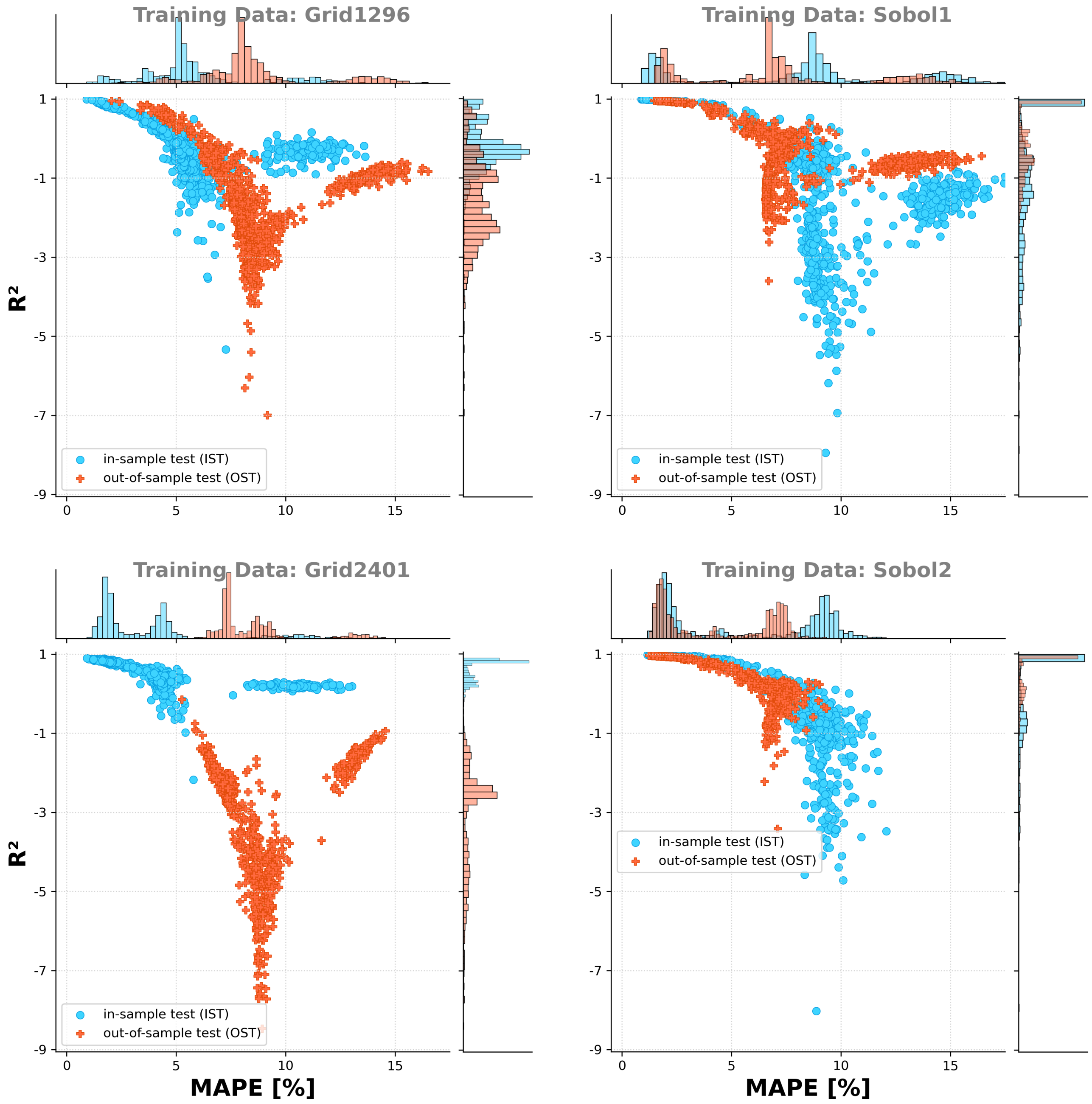

Figure 30. R^2 scores plotted against the MAPE of the neural network regression models. Comparing the OST (orange) to the IST (blue) the, models trained using the Grid1296 and Grid2401 (first column) perform noticeably worse than the models trained based on the Sobol1 and Sobol2 datasets (second column). The histograms above and on the right show the distribution of the MAPE and R^2, respectively.

Table 17. The 12 FNN models with the lowest MAPE and R^2 score, respectively, that are used to substitute the MD simulations in the SMAOpts. Shown are the ratio to divide the dataset into training and test data and the random seed that is used for this split. The ID is a consecutive number showing that some of the models belong to both, the top 12 minMAPE and top 12 maxR^2 group, resulting in 17 different SMAOpts instead of 24. The models with the "SMAOpt-1" and "SMAOpt-2" remark are the models used in the optimization runs with the corresponding names.

minMAPE	Split Ratio	Rnd Seed	Dataset	MAPE	R²	ID	Opt. Run Ref.
1	0.95	951	Sobol1	0.0088	0.9924	1	
2	0.85	222	Sobol1	0.0090	0.9934	2	
3	0.95	999	Sobol1	0.0095	0.9926	3	
4	0.95	222	Sobol1	0.0095	0.9940	4	
5	0.95	490	Sobol1	0.0095	0.9933	5	
6	0.95	171	Sobol1	0.0096	0.9896	6	
7	0.85	877	Sobol1	0.0100	0.9927	7	
**8**	**0.95**	**986**	**Sobol1**	**0.0101**	**0.9919**	**8**	**SMAOpt-1**
9	0.85	669	Sobol1	0.0102	0.9909	9	
10	0.95	561	Sobol1	0.0103	0.9906	10	
**11**	**0.95**	**928**	**Sobol1**	**0.0104**	**0.9901**	**11**	**SMAOpt-2**
12	0.85	633	Sobol1	0.0104	0.9899	12	
**maxR^2**	**Split Ratio**	**Rnd Seed**	**Dataset**	**MAPE**	**R²**	**ID**	**Opt. Run Ref.**
1	0.95	222	Sobol1	0.0095	0.9940	4	
2	0.85	222	Sobol1	0.0090	0.9934	2	
3	0.95	490	Sobol1	0.0095	0.9933	5	
4	0.85	877	Sobol1	0.0100	0.9927	6	
5	0.85	78	Sobol1	0.0106	0.9926	13	
6	0.95	999	Sobol1	0.0095	0.9926	3	
7	0.95	968	Sobol1	0.0106	0.9924	14	
8	0.95	951	Sobol1	0.0088	0.9924	1	
9	0.85	249	Sobol1	0.0111	0.9923	15	
**10**	**0.95**	**986**	**Sobol1**	**0.0101**	**0.9919**	8	**SMAOpt-1**
11	0.85	936	Sobol1	0.0112	0.9915	16	
12	0.95	634	Sobol1	0.0104	0.9910	17	

Model Selection

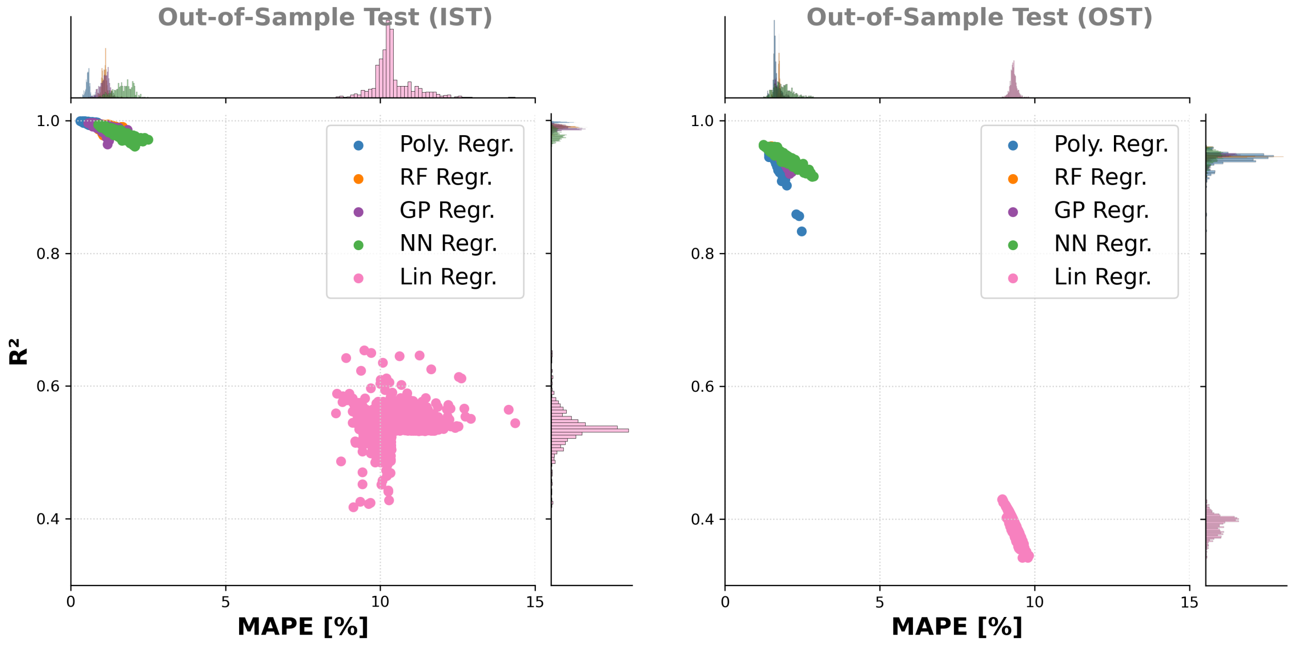

Figure 31. Overview of the models with the 500 best mean absolute percentage error (MAPE) and best 500 coefficient of determination (R^2) scores including the linear regression models. The left graph shows the MAPE vs R^2 score regarding to the in-sample test (IST), while the right graph shows these values w.r.t. to the out-of-sample test (OST). Note that models that are part of the top 500 MAPE and top 500 R^2 score groups are plotted once, not twice. The histograms above and on the right show the distribution of the MAPE and R^2, respectively.
